# Long/post-COVID in children and adolescents: symptom onset and recovery after one year based on healthcare records in Germany

**DOI:** 10.1007/s15010-024-02394-8

**Published:** 2024-09-16

**Authors:** Franz Ehm, Falko Tesch, Simone Menzer, Friedrich Loser, Lars Bechmann, Annika Vivirito, Danny Wende, Manuel Batram, Tilo Buschmann, Marion Ludwig, Martin Roessler, Martin Seifert, Giselle Sarganas Margolis, Lukas Reitzle, Christina Koenig, Claudia Schulte, Pedro Ballesteros, Stefan Bassler, Thomas Bitterer, Cordula Riederer, Reinhard Berner, Christa Scheidt-Nave, Jochen Schmitt, Nicole Toepfner

**Affiliations:** 1 Center for Evidence-Based Healthcare (ZEGV), University Hospital and Faculty of Medicine Carl Gustav Carus, TU Dresden, Fetscherstraße 74, 01307 Dresden, Germany; 2 IKK Classic, Tannenstraße 4 B, 01099 Dresden, Germany; 3 Techniker Krankenkasse, Bramfelder Straße 140, 22305 Hamburg, Germany; 4 InGef - Institute for Applied Health Research Berlin GmbH, Otto-Ostrowski-Straße 5, 10249 Berlin, Germany; 5 BARMER Institut für Gesundheitssystemforschung (Bifg), Axel-Springer-Straße 44, 10969 Berlin, Germany; 6 Vandage GmbH, Detmolder Str. 30, 33604 Bielefeld, Germany; 7 AOK PLUS, Sternplatz 7, 01067 Dresden, Germany; 8 Robert Koch Institute, Nordufer 20, 13353 Berlin, Germany; 9 DAK-Gesundheit, Nagelsweg 27 – 31, 20097 Hamburg, Germany; 10 Department of Pediatrics, University Hospital and Faculty of Medicine Carl Gustav Carus, TU Dresden, Fetscherstraße 74, 01307 Dresden, Germany

**Keywords:** COVID-19, Post-COVID, Electronic health records, Epidemiology, Pediatrics

## Abstract

**Purpose:**

Evidence on the incidence and persistence of post-acute sequelae of COVID-19 (PASC) among children and adolescents is still limited.

**Methods:**

In this retrospective cohort study, 59,339 children and adolescents with laboratory-confirmed COVID-19 in 2020 and 170,940 matched controls were followed until 2021-09-30 using German routine healthcare data. Incidence rate differences (ΔIR) and ratios (IRR) of 96 potential PASC were estimated using Poisson regression. Analyses were stratified according to age (0–11, 12–17 years), and sex. At the individual level, persistence of diagnoses in patients with onset symptoms was tracked starting from the first quarter post-infection.

**Results:**

At 0–3 month follow-up, children and adolescents with a previous SARS-CoV-2 infection showed a 34% increased risk of adverse health outcome, and approximately 6% suffered from PASC in association with COVID-19. The attributable risk was higher among adolescents (≥ 12 years) than among children. For most common symptoms, IRRs largely persisted at 9–12 month follow-up. IRR were highest for rare conditions strongly associated with COVID-19, particularly inflammatory conditions among children 0–11 years, and chronic fatigue and respiratory insufficiency among adolescents. Tracking of diagnoses at the individual level revealed similar rates in the decline of symptoms among COVID-19 and control cohorts, generally leaving less than 10% of the patients with persistent diagnoses after 12 months.

**Conclusion:**

Although very few patients presented symptoms for longer than 12 months, excess morbidity among children and, particularly, adolescents with a history of COVID-19 means a relevant burden for pediatric care.

**Supplementary Information:**

The online version contains supplementary material available at 10.1007/s15010-024-02394-8.

## Introduction

Children and adolescents are more likely to be asymptomatic or develop a mild illness following SARS-CoV-2 infection compared to adults [1–3]. However, a small percentage of the infected children and adolescents show symptoms beyond the acute phase of COVID-19. Various terms have been proposed to denote long-term otherwise unexplained health problems following SARS-CoV-2 infection [4–8]. Long-COVID has been widely used to denote symptoms that have not resolved within four weeks following acute infection or newly occurred thereafter. The term post-acute health sequelae of COVID-19 (PASC) has been frequently used in a similar sense, referring to adverse health outcomes manifesting beyond the acute phase of infection. Post-COVID-19 syndrome (PCS) [4] or post-COVID condition (PCC) [3] refer to persistent, new-onset, or recurrent health complaints present after 12 weeks and beyond. These terms, and several variations, are used interchangeably, too [9–11]. Diagnosis of PASC and PCS is challenged by inter-individual heterogeneity and the lack of specific diagnostic markers [3, 8, 12]. The use of different outcome definitions in epidemiological studies, the occurrence of symptoms in control subjects, and the differentiation of PASC from indirect health consequences of the pandemic [13, 15] affect scientific evidence on long/post-COVID in children and adolescents. Hence, prevalence rates estimates of PASC and PCS in children and adolescents have varied substantially across different studies as pointed out by several systematic reviews [2, 11, 16–18]. While one meta-analysis presented a pooled prevalence of 25% [16], other systematic reviews demonstrated that in controlled studies the actual differences in incidence of PASC between COVID-19 and control group were actually lower in the range from 1 to 5% [2, 17].

To address the lack of a commonly accepted definition of long/post-COVID in children and adolescents, a core outcome set of symptoms (PC-COS) was still being elaborated at the time of this study, and finally published in February 2024 [19]. Previously, the WHO had released a case definition of PCC in children and adolescents in February 2023 [3]. In total, the WHO consensus cited 21 different symptoms with fatigue, altered smell/taste, and anxiety being the most strongly associated with PCC. Furthermore, a rare but life-threatening condition was reported up to six weeks following SARS-CoV-2 infection in the form of multisystem inflammatory syndrome in children (MIS-C), involving different body parts and often requiring treatment in a hospital [20, 21].

Several studies have investigated the persistence of symptoms in association with long/post-COVID. The CLoCk study revealed fatigue and shortness of breath as relevant PASC after six months, being reported substantially more often in the group of children with previous COVID-19 (9.5% versus 1.2% and 3.9% versus 0.4% for controls, respectively) [22]. After 12 months of follow-up, general symptoms were about 20% more likely among children with previous infection compared to controls [23]. In a cross-sectional population-based study from Denmark, adolescents with SARS-CoV-2 infection reported more long-lasting symptoms and had longer sick leave compared to controls [14]. A Norwegian study with routine data showed that increased primary care utilization following COVID-19 was longer observed in preschool-aged children (up to six months) than in primary or secondary school students (up to three months) [24]. Several meta-analyses of the symptom burden from PASC over time emphasized that data for children are still limited and especially age-stratified analysis is needed to better understand the different patterns of symptom persistence in children and adolescents [16, 25]. Contradicting evidence of early symptom resolution and prolonged or late excess morbidity after infection motivates more research on the persistence of long/post-COVID at both the population level and individual level [16, 17].

Our primary study objective was therefore to identify long-term morbidity patterns in association with COVID-19, distinguishing the effects on young children aged 0–11 years, and adolescents aged 12–17 years. Secondary objective was to describe persistence and changes of these patterns over the course of one year at the population level and individual level. The tertiary objective was to compare our findings to the expert consensus on PCC in children/adolescents released by the WHO in 2023. Based on diagnoses of pediatricians and general practitioners of in total 6,279,522 children and adolescents, we aim to enhance and integrate evidence on pediatric COVID-19 sequelae derived from routine healthcare data into current concepts of PCC.

## Methods

### Study design and data

We designed a retrospective matched cohort study based on routine healthcare data covering about half of the German pediatric population. Analysis was conducted as part of the Post-COVID-19 Monitoring in Routine Health Insurance Data (POINTED) program involving five German healthcare insurance companies, and three research institutes. The POINTED methodology was described in detail in our previous work [26]. The present study included anonymized inpatient and outpatient healthcare data documented between January 1, 2019, and September 30, 2021. All data were harmonized and aggregated from the different data owners using a coordinated process and synchronized routines coded in R, thereby meeting the requirements of German data protection regulations. We specifically used available information on age and sex, vital status (date of death), medical diagnoses as documented in medical records according to the International Classification of Diseases and Related Health (ICD-10-GM), inpatient and outpatient medical procedures, and prescription medication. The POINTED study protocol was approved (approval number: BO-EK (COVID)-482,102,021) by the ethics committee of the TU Dresden (IRB00001473) and adheres to all relevant administrative and legal regulations. The study was registered at ClinicalTrials.gov (NCT number: NCT05074953). The presentation of methods and results was informed by the RECORD Statement for cohort studies using routine healthcare data [27].

### COVID-19 and control cohorts

**Fig. 1:**
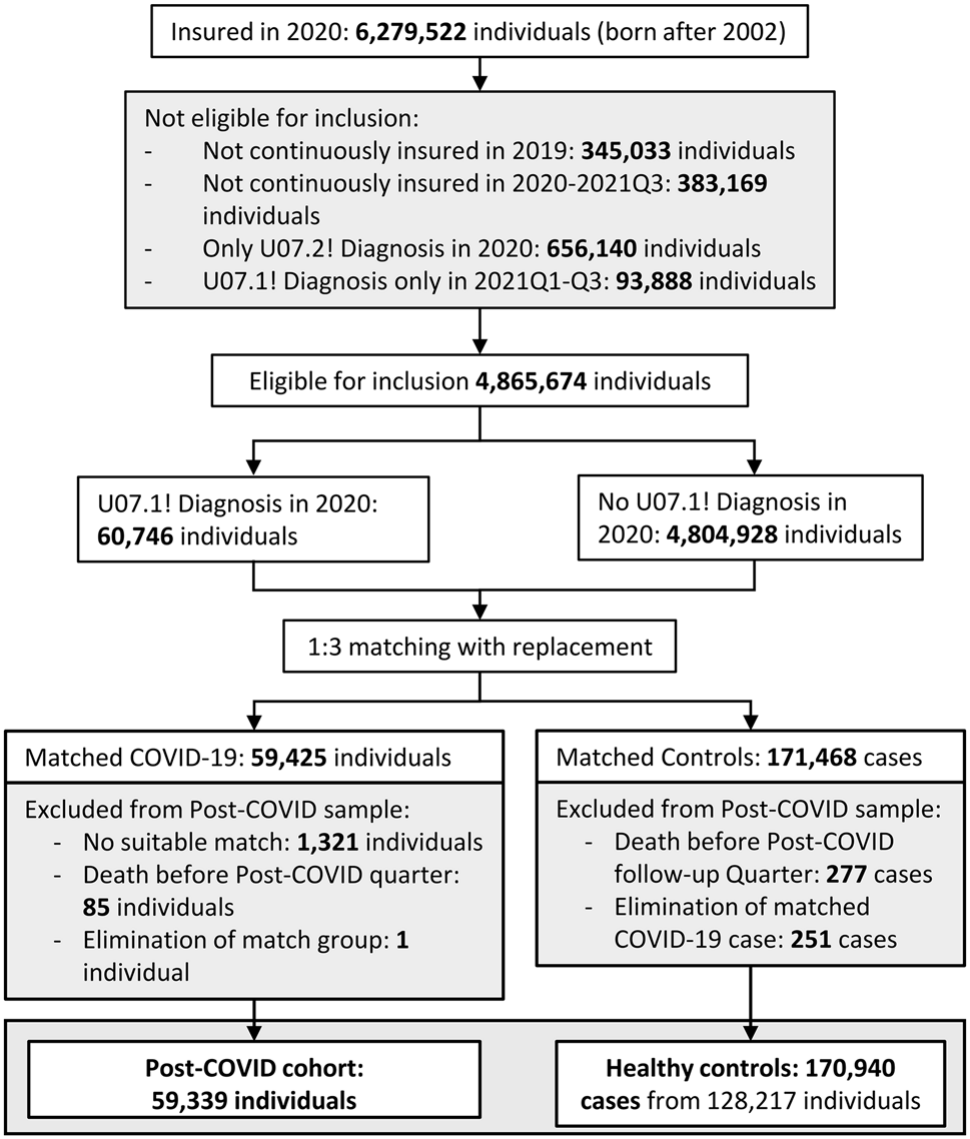
Flowchart of children and adolescents included in the analysis

From the 6,279,522 persons aged under 18 years and being insured at least one day in 2020, we included 59,339 COVID-19 cases and 170,940 controls in our study cohort (Fig. 1). COVID-19 cases were included for analysis based on Polymerase-chain-reaction (PCR) confirmed diagnoses of SARS-CoV-2 infection (ICD-10: U07.1!) between January 1 and December 31, 2020 and followed up until September 30, 2021. To account for individual differences in the follow-up time we calculated the case-specific time-at-risk between index and end date (death or end of insurance before September 30, 2021). Children and adolescents who had never tested positive for SARS-CoV-2 infection were considered as controls. To control for confounding and enable stratified analysis, we matched each COVID-19 case to three controls based on exactly the same age and sex, and using a propensity score from healthcare utilization and 14 comorbidities in the pre-observational period [26].

### Health outcomes

Based on previous work within the POINTED group, we examined potential PASC by 96 different health outcomes, 13 diagnostic complexes, and three overarching health domains [26]. Each outcome is related to one or multiple ICD-10 codes, occasionally subsuming rare conditions (as in inflammatory disorders). Two additional control endpoints, prescription glasses and acne, were defined to control results on the association between health outcomes and COVID-19 for potential detection bias. Furthermore, we studied PCC as the presence of at least one of the three most specific conditions cited by the WHO (fatigue, altered smell/taste, and anxiety). [3] A complete list of (grouped) diagnoses including ICD-10 codes is given in Supplementary Tables S8-S10.

### Statistical analyses

To quantify the excess risk of health outcomes following COVID-19 we considered absolute differences in incidence rates (ΔIR) and incidence rate ratios (IRR) estimated via Poisson models with time-at-risk as offset. The reasons for using Robust Poisson regression on aggregate data were elaborated previously [26]. Incidence rates (IR) were calculated per 1000 person-years. For each symptom under study, we assessed incident events among the population at risk for this outcome excluding prevalent cases with at least one documented diagnosis in the pre-observational period. As a result of the quarterwise billing in the German statutory health care system, observational periods were also denoted quarterwise. We refer to the first quarter Q1 after the index quarter as a follow-up of ‘0–3 months’. In fact, Q1 included diagnoses within at least three months after COVID-19 and up to almost six months after COVID-19 depending on the actual date of index. Hence, Q1 was considered the first quarter to study the onset PCC in children.

At the population level, the persistence of health outcomes was evaluated by comparing IRRs and ΔIRs after 0–3 months and 9–12 months of follow-up. Stratified analyses were conducted considering age and sex. At the individual level, the persistence of symptoms was measured for each COVID-19 and control case by tracking onset diagnoses starting from Q1. A symptom/cluster was considered persistent if it was successively diagnosed throughout the follow-up period (with a maximum of one intermediate quarter of non-diagnosis). Analyses were coded in R (version 3.6.3) using common software libraries for data analysis and statistics.

## Results

### Descriptives of the COVID-19 and control cohorts

Table 1 summarizes the descriptive characteristics of the 59,399 children with a documented SARS-CoV-2 infection in 2020 and the 170,940 matched controls. Almost 99% of the COVID-19 cases were documented in an outpatient setting.

**Table 1:** Descriptive summary of COVID-19 and control cohort by age, sex, severity of COVID-19

**a** Characteristics of the cohort				
	COVID-19 N (%)		Control N (%)	
Total	59,339	(100%)	170,940	(100%)
Age				
0–11 years	33,201	(56.0%)	96,071	(56.2%)
12–17 years	26,138	(44.0%)	74,869	(43.8%)
Sex				
Male	30,435	(51.3%)	87,623	(51.3%)
Female	28,904	(48.7%)	83,317	(48.7%)
Severity of COVID-19				
Outpatient	58,437	(98.5%)	–	–
Hospital	717	(1.2%)	–	–
ICU	185	(0.3%)	–	–
Observable cases in follow-up quarter				
Q1 (0–3mos)	59,339	(100%)	170,940	(100%)
Q2 (3–6mos)	59,339	(100%)	170,940	(100%)
Q3 (6–9mos)	59,243	(99.8%)	170,378	(99.7%)
Q4 (9–12mos)	31,236	(52.6%)	90,162	(52.7%)
Q5 (12–15mos)	14,763	(24.9%)	42,546	(24.9%)
Q6 (15–18mos)	4,984	(8.4%)	14,386	(8.4%)
Mean time-at-risk per quarter	0.365 years		0.365 years	

**b** Mean values of covariates		
Statistic (based on the four quarters preceding the index)	COVID-19	Control
General characteristics		
Mean age [years]	9.18	9.19
Quarters with outpatient care	2.93	2.92
Received any inpatient care [%]	10.0%	10.2%
Comorbidities*		
Asthma	1.2%	1.2%
Autoimmune disorder	7.4%	7.3%
Bronchopulmonary dysplasia	0.0%	0.0%
Cancer	0.1%	0.2%
Congenital heart disease	1.4%	1.5%
Diabetes (other) with insulin	0.1%	0.1%
Dialysis	0.0%	0.0%
Down syndrome	0.4%	0.1%
Epilepsy	0.8%	0.9%
Immunodeficiency (primary)	0.5%	0.5%
Immunosuppressive disease	0.9%	0.9%
Immunosuppressive therapy	0.2%	0.2%
Obesity	0.1%	0.1%
Psychomotor disorder	2.5%	2.7%

*Definitions are given in supplementary table S12

The majority of the SARS-CoV-2 infections took place in the last quarter of 2020, meaning only a few patients were observable for more than four quarters after the index. The matched groups were well balanced for the considered comorbidities as was the amount of healthcare utilization.

### Overview of relevant health outcomes at three and twelve months follow-up

**Fig. 2:**
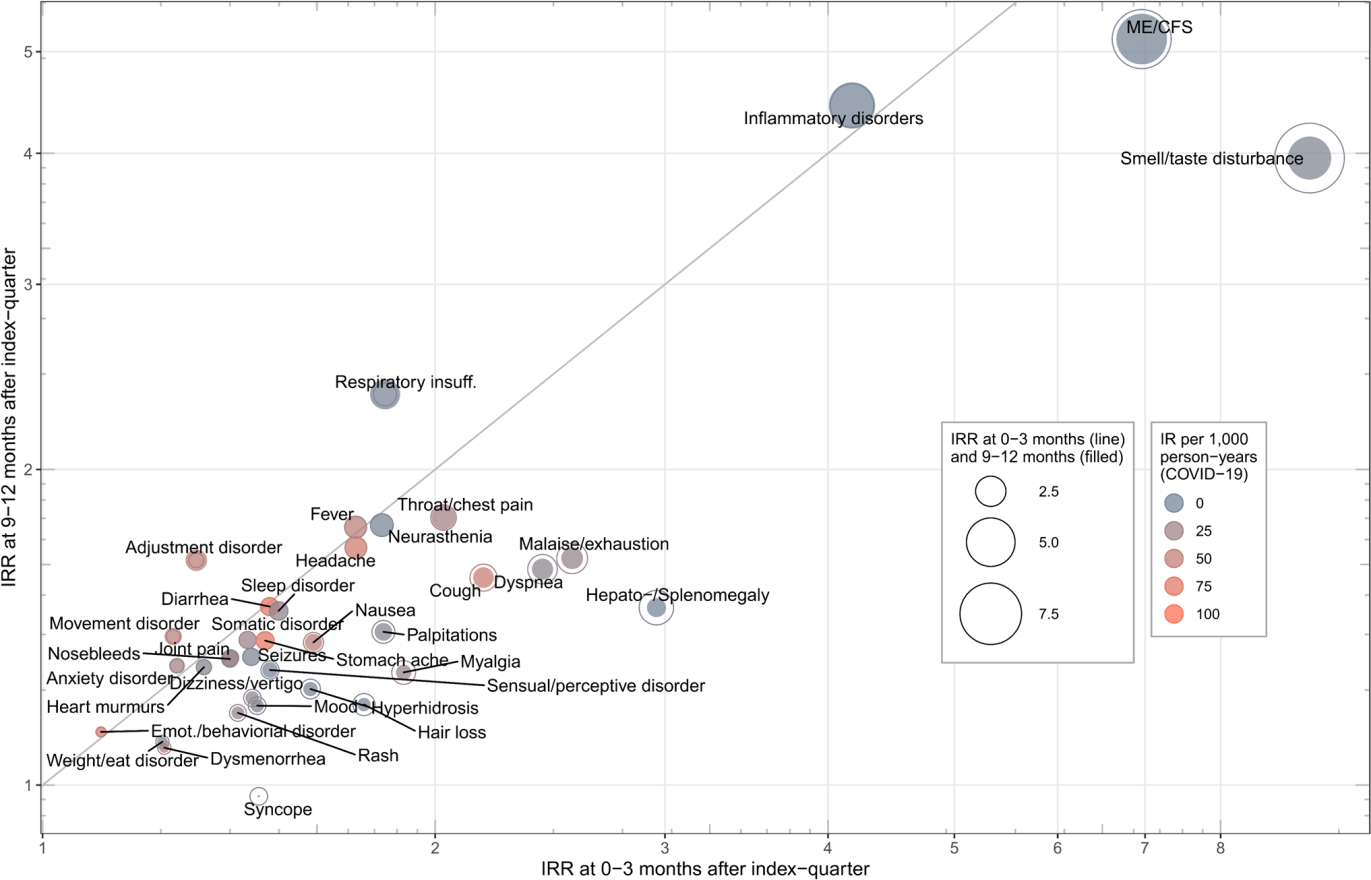
Relative risk of studied symptoms and conditions after three and 12 months. The figure shows symptoms and conditions with significant excess relative risk at three months after acute COVID-19 (i.e. health outcomes with a lower limit of CI 95% greater than 1 in the first and second quarter after index). Coordinates refer to the IRR after the first, and the fourth quarter of follow-up. For each symptom, dot size is proportional to the magnitude of relative risk after three months (line) and after 12 months (filled), respectively. Different colors are used to denote the frequency in terms of IR of an onset health condition in the COVID-19 group. Conditions positioned below (above) the diagonal line indicate a decreasing (increasing) impact of COVID-19 on incident diagnoses over the time course. Note: Due to very small number of diagnoses for Chronic fatigue (ME/CFS) in the COVID-19 and control group we could not calculate the point estimate after 9–12 months. In this circumstance, the IRR shown refers to 6–9 months (graphic created using the ggplot2 package in R)

Considering the overall health outcome defined by any of the 96 endpoints, we found an absolute risk difference ΔIR of 155 per 1000 person-years (IR COVID-19: 603.99, IR control: 449.40) in the first 0–3 months after the index quarter. At this stage of follow-up, the estimated relative risk of suffering from any of the 96 symptoms following COVID-19 was 1.34 (CI 95% 1.31, 1.38). Figure 2 provides an overview of the subset of individual symptoms showing significant excess relative risk in the post-acute phase of COVID-19. Frequent diagnoses in the COVID-19 group included stomachache, headache, cough, diarrhea, throat/chest pain, and fever. The position of most of these general symptoms close to the diagonal line implies consistent relative risk over time. High IRRs were found for less frequent conditions typically reported in association with PCS or PCC such as smell/taste disturbance, chronic fatigue syndrome (ME/CFS), malaise/exhaustion, and dyspnea. We observed a relative decline in the onset of many PASC in the COVID-19 group and thus decreasing IRRs after 12 months, yet the excess relative risk of these symptoms remained high. The more detailed view in Supplementary Fig. 1 reveals a general pattern of growing incidence over time for the majority of symptoms in both groups.

### Incidence of health outcomes by age group

Table 2 shows the separate rankings of the five symptoms with highest ΔIR and highest IRR in children and adolescents after 0–3 months and 9–12 months, respectively.

**Table 2:** Top five ranked outcomes with highest incidence rate difference (ΔIR) and with highest incidence rate ratio (IRR) after 0–3 months and after 9–12 months for children and adolescents

**a** Children aged 0–11 years	0–3 months follow up				9–12 months follow up			
	Rank	IR COVID	IR Control	IRR (95% CI)	Rank	IR COVID	IR Control	IRR (95% CI)
Top 5 ΔIR								
Cough	1	45.35	22.71	2.00 (1.71, 2.33)	3	64.77	43.95	1.47 (1.26, 1.72)
Stomachache	2	59.43	39.79	1.49 (1.32, 1.69)	4	60.97	44.59	1.37 (1.17, 1.60)
Diarrhea	3	47.04	30.72	1.53 (1.33, 1.77)	1	76.75	52.99	1.45 (1.25, 1.68)
Fever	4	37.29	23.57	1.58 (1.35, 1.86)	2	58.36	34.73	1.68 (1.41, 2.00)
Headache	5	26.71	15.13	1.77 (1.47, 2.12)	–	33.62	18.77	1.79 (1.44, 2.23)
Movement disorder	–	39.17	31.78	1.23 (1.07, 1.42)	5	52.48	36.41	1.44 (1.22, 1.70)
Top 5 IRR								
Smell/taste disturbance	1	3.54	0.40	8.90 (3.47, 22.78)	2	3.58	0.86	4.16 (1.71, 10.15)
Inflammatory disorders	2	1.90	0.32	6.00 (2.03, 17.69)	1	1.87	0.41	4.59 (1.27, 16.58)
Malaise/exhaustion	3	11.51	4.05	2.85 (2.05, 3.95)	3	10.76	5.72	1.88 (1.28, 2.77)
Cough	4	45.35	22.71	2.00 (1.71, 2.33)	–	64.77	43.95	1.47 (1.26, 1.72)
Myalgia	5	13.86	7.38	1.88 (1.45, 2.43)	–	15.36	11.47	1.34 (1.00, 1.80)
Headache	–	26.71	15.13	1.77 (1.47, 2.12)	4	33.62	18.77	1.79 (1.44, 2.23)
Somatic disorder	–	13.91	9.15	1.52 (1.19, 1.94)	5	18.22	10.19	1.79 (1.33, 2.40)
Control outcomes								
Acne		4.65	4.73	0.98 (0.68, 1.42)		4.77	5.60	0.85 (0.54, 1.36)
Prescription glasses		31.45	29.68	1.06 (0.90, 1.24)		34.88	31.90	1.09 (0.90, 1.33)

**b** Adolescents aged 12–17 years	0–3 months follow up				9–12 months follow up			
	Rank	IR COVID	IR Control	IRR (95% CI)	Rank	IR COVID	IR Control	IRR (95% CI)
Top 5 ΔIR								
Headache	1	67.31	39.03	1.72 (1.51, 1.97)	1	103.96	63.79	1.63 (1.39, 1.91)
Stomachache	2	79.02	53.80	1.47 (1.30, 1.66)	3	100.49	72.83	1.38 (1.18, 1.62)
Malaise/exhaustion	3	33.01	13.58	2.43 (1.98, 2.99)	–	33.09	21.44	1.54 (1.19, 2.01)
Throat/chest pain	4	33.04	14.49	2.28 (1.86, 2.80)	4	44.74	23.02	1.94 (1.52, 2.49)
Dyspnea	5	25.53	8.62	2.96 (2.30, 3.81)	–	19.68	10.94	1.80 (1.26, 2.57)
Diarrhea	–	49.74	34.26	1.45 (1.25, 1.69)	2	90.20	59.23	1.52 (1.28, 1.81)
Cough	–	21.51	7.67	2.80 (2.13, 3.69)	5	35.82	18.49	1.94 (1.47, 2.56)
Top 5 IRR								
Smell/taste disturbance	1	15.95	1.68	9.50 (5.67, 15.90)	2	9.40	2.45	3.84 (1.96, 7.54)
Chronic fatigue syndrome	2	2.52	0.37	6.86 (2.23, 21.04)	–	0.46	0.61	0.75 (0.12, 4.69)
Respiratory insufficiency	3	1.89	0.54	3.48 (1.31, 9.27)	1	3.44	0.61	5.62 (1.53, 20.69)
Hepato-/Splenomegaly	4	2.84	0.82	3.45 (1.56, 7.64)	–	2.53	1.57	1.61 (0.62, 4.18)
Dyspnea	5	25.53	8.62	2.96 (2.30, 3.81)	–	19.68	10.94	1.80 (1.26, 2.57)
Cough	–	21.51	7.67	2.80 (2.13, 3.69)	5	35.82	18.49	1.94 (1.47, 2.56)
Fever	–	12.55	4.87	2.57 (1.82, 3.64)	3	19.52	8.88	2.20 (1.49, 3.25)
Throat/chest pain	–	33.04	14.49	2.28 (1.86, 2.80)	4	44.74	23.02	1.94 (1.52, 2.49)
Control outcomes								
Acne		72.31	66.31	1.09 (0.97, 1.22)		86.79	84.52	1.30 (0.88, 1.20)
Prescription glasses		51.44	52.66	0.98 (0.85, 1.13)		36.30	34.87	1.04 (0.81, 1.33)

Only outcomes with significant IRR in at least the first follow-up quarter are shown. Incidence rates (IR) are calculated per 1000 person-years. WHO definition of PCC includes smell/taste disturbance, chronic fatigue, malhaise/exhaustion and anxiety disorder. ‘Inflammatory disorders’ include diseases with systemic inflammatory processes such as MIS-C, PIMS, SIRS, and juvenile rheumatoid arthritis with systemic onset

First, we found common symptoms such as cough, stomachache, fever, headache, and diarrhea as relevant outcomes with high absolute risk both in young children and adolescents. IRR of most of these conditions ranged from 1.5 to 2 and were generally unchanged at 9–12 months of observation time.

Second, we found high IRRs after 0–3 months for conditions typically described in association with COVID-19. Most notably, smell or taste disturbances were detected almost equally in younger children (IRR: 8.90, 95% CI 3.47–22.78) and adolescents (IRR: 9.50, 95% CI 5.67–15.90). Conditions of fatigue were considerably more frequent in the COVID-19 group. Malaise/exhaustion showed a slightly higher IRR in the COVID-19 group of adolescents aged 12–17 years (IRR: 2.85 95 CI 2.05–3.95) compared to children aged 0–11 years (IRR: 2.43, 95% CI 1.98–2.99). Considering myalgic encephalomyelitis/chronic fatigue syndrome (ME/CFS) as a serious and potentially long-lasting form of fatigue, our results show a significant risk increase following COVID-19 in adolescents (IRR: 6.86, 95% CI 2.23–21.04). Such diagnoses were generally more frequent in adolescents than in young children. Despite a similar pattern of decreasing relative risk in both age groups, IRRs for these symptoms remained high after 12 months.

Third, we found age-group-specific differences in particular health outcomes. Respiratory problems were more frequent in adolescents aged 12 years or older. In this age group, a high number of onset diagnoses for dyspnea, cough, and respiratory insufficiency was observed in the first quarter of follow-up. The relative risk for respiratory insufficiency even increased after 9–12 months (IRR: 5.62, 95% CI 1.53–20.69). We further noted hepatomegaly and splenomegaly among the most frequently diagnosed conditions in adolescents following COVID-19 showing an IRR of 3.45 (95% CI 1.56–7.64) after 0–3 months. Observing the characteristic symptoms in children aged 0–11 years, inflammatory disorders were striking. Without discerning underlying mechanisms of early and late inflammatory response in the conditions subsumed by this outcome, significant excess risk is found throughout all four follow-up quarters (IRR: 6.00, 95% CI 2.03–17.69 after 0–3 months, IRR: 4.59, 95% CI 1.27–16.58 after 9–12 months).

### Control endpoints

To control for effects of detection bias from COVID-19 we estimated IRRs for the prescription of glasses and for acne. As listed in Table 2, there was no statistical association of these outcomes with COVID-19, with IRRs close to 1.

### Diagnostic complexes

Following COVID-19, adolescents showed higher absolute and relative risk across symptom complexes compared with younger children (Supplementary Table S1). For most complexes, this additional risk narrowed in both age groups with IRRs remaining elevated in the range of 1.3–1.5 after 9–12 months. The highest IRR was found for pulmonary symptoms in adolescents (IRR: 2.85, 95% CI 2.37–3.42). This is in line with our findings for separate respiratory symptoms. Absolute excess risk was highest for neuropsychiatric symptoms with ΔIRs close to 50 (30) per 1000 person-years, and gastrointestinal symptoms with ΔIRs of 41 (35) per 1000 person-years in adolescents (children). Stratified analysis for female and male sex revealed no relevant differences in the IRR estimates of health domains (physical, mental, overlap) and diagnostic complexes. However, absolute incidence was generally higher among girls (Supplementary tables S4 and S5).

### Persistence of symptoms at the individual level

**Fig. 3:**
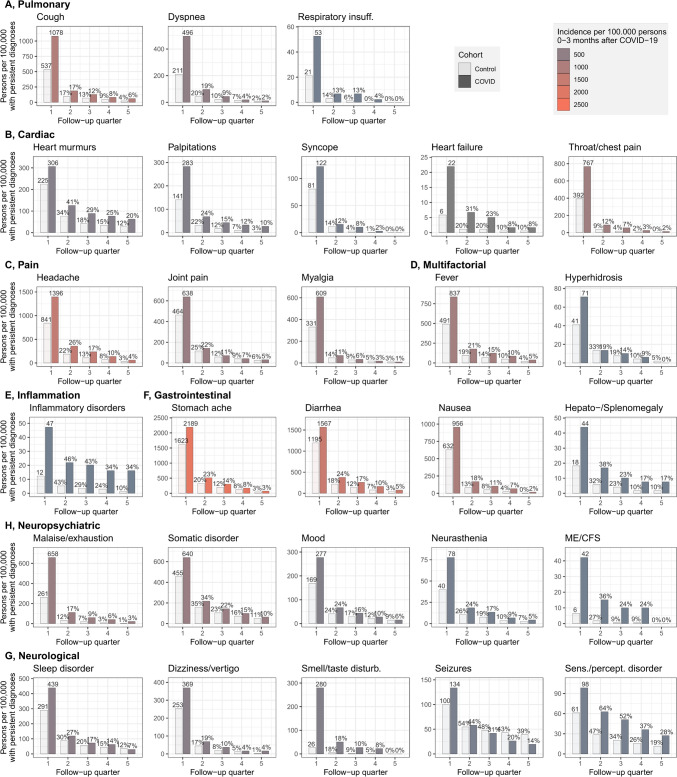
Persistence of conditions in individuals in the COVID-19, and in the control group with an onset diagnosis in the first follow-up quarter. Shown are conditions from relevant symptom complexes with an IRR $\ge$ 1.3 in the 0–3 months after the index quarter. Persistence is measured by tracking repeated occurrence of a diagnosis in subsequent follow-up quarters with at most one quarter missing between two diagnoses. Persons who are no more observable are censored in the analysis and persistence is calculated by the share of repeated diagnoses after censoring (graphic created using the ggplot2 package in R)

Figure 3 contains persistence plots for onset symptoms with significant excess relative risk in the first quarter of follow-up (compare Fig. 2). For the vast majority of symptoms, the share of persistent cases diminished clearly after six months and was reduced to below 10% after one year of follow-up. Persistence rates were mostly similar for COVID-19 and control patients. Conditions showing faster recovery in the COVID-19 group with the number of cases ultimately converging between the groups included myalgia, joint pain, sleep disorders, mood swings, and hyperhidrosis. Several conditions were more persistent in the COVID-19 group such as cardiac and gastrointestinal symptoms, inflammatory disorders, respiratory insufficiency, fatigue (malaise/exhaustion or ME/CFS), and sensation/perception disorder.

### Comparison to WHO PCC consensus

Within 0–3 months following the index quarter, patients with COVID-19 had a two times higher risk of suffering from any of the three potential PCC diagnoses considered as particularly relevant in the WHO 2023 consensus on PCC in children and adolescents. The excess incidence in the COVID-19 group during this period was 24.6 per 1000 person-years (Supplementary Table S3). Among the symptoms included in the WHO definition, anxiety stood out with an IRR of 1.27 (CI 95% 1.09, 1.48). Analysis excluding anxiety revealed a considerably higher IRR of 3.29 (CI 95% 2.81, 3.85) while maintaining a comparable ΔIR of 21.1 per 1000 person-years with adolescents more frequently affected than children (34.4 versus 10.7 per 1000 person-years). After 9–12 months, IRR and ΔIR decreased by about 50% in adolescents and 30% in young children.

Girls were generally more frequently diagnosed with one of the four PCC related health outcomes, whereas boys had a slightly higher relative risk (Supplementary Table S6).

## Discussion

### Interpretation of results

The present study revealed an estimated 34% excess relative risk and an attributable risk of 155 per 1000 person-years for children and adolescents with documented SARS-CoV-2 infection in 2020 to suffer from unspecific PASC within the first three months after the index quarter. Considering a mean time-at-risk of 0.365 years per person during this period, this means 5–6% of the children and adolescents with a documented SARS-CoV-2 infection showed onset symptoms that are statistically attributable to COVID-19. The relative risk in the COVID-19 group to suffer from specific PASC such as smell/taste disturbance, and fatigue was almost 330% corresponding to an excess incidence of 21 per 1000 person-years (Supplementary Table S3). Put differently, almost 1% of the children with a documented SARS-CoV-2 infection had an onset diagnosis of smell/taste disturbance (0.3%) or fatigue (0.5%) attributable to COVID-19.

At 9–12 months of follow-up, IRRs for many common unspecific symptoms remained close to 1.5, which hints at changes in the immune response toward other infectious diseases following COVID-19. In line with other studies, we found a considerable drop in the relative risk of COVID-19-specific symptoms after one year. We noted a general pattern of increasing incidence over time for almost all health conditions in the control group as visualized in Supplementary Fig. 1. This could be attributed to the (accumulating) baseline risk of symptom onset in non-prevalent cases over time. In the COVID-19 group, this pattern only applied to unspecific conditions.

Age-stratified results suggested that adolescents were more affected by PCC-specific symptoms. Most likely, they can describe their symptoms much more elaborately than younger children. Adolescents were also more affected by respiratory problems following COVID-19 compared to children. Furthermore, we noticed a shift in the excess incidence of diagnoses after 0–3 months versus after 9–12 months pointing towards an aggravation of respiratory symptoms in many adolescent patients, e.g. ΔIR of dyspnea decreased by almost 50% (16.9 versus 8.7 per 1000 person-years) whereas ΔIR of respiratory insufficiency doubled (1.4 versus 2.8 per 1000 person-years).

We found a particular increase in the risk of inflammatory disorders in children. We suppose there are different aspects of inflammatory processes represented in our estimates reflecting immediate immune response (e.g. PIMS/MIS-C) as well as delayed immune response (e.g. juvenile arthritis). COVID-19 as a potential driver of inflammatory (auto-) immune response is broadly discussed. Previous electronic health records-based studies reported an excess risk of 20–40% for onset autoimmune disease after COVID-19 [28, 29].

ME/CFS is a serious disabling chronic disease. Patients suffer from overwhelming fatigue, which is not improved by rest and worsens after any kind of physical or mental activity. Current hypotheses on the interference of ME/CFS and COVID-19 include the possible exacerbation of latent pathogenic factors following acute COVID-19, or particular manifestations of PCC over the course of 6 months meeting the criteria of ME/CFS [30]. Both hypotheses are supported by our analyses. We found very high effects for ME/CFS at 0–3 months after the index quarter, albeit with broad confidence intervals especially in younger children (IRR: 7.50, 95% CI 0.59, 96.58). While we found significant excess relative risk in adolescents after 6–9 months (IRR: 4.80, 95% CI 1.23, 18.74), estimating the risk beyond 9 months proved unrealistic due to the limited number of observable cases at this point.

Following individuals with onset PASC from quarter 1 to quarter 5 after the index, we found most conditions showed similar persistence rates in the COVID-19 and control cohorts. For the majority of incident health outcomes our findings suggest a similar prognosis regarding symptom resolution in both study groups, although elevated persistence rates among COVID-19 patients were visible for some diagnoses (discussed below). From the patients with incident health outcomes, only a minority showed symptoms persisting for more than one quarter. Following patients for a further two quarters (6–9 months), and three quarters (9–12 months) after the first quarter with an onset diagnosis reduced the persistence rates of most outcomes to under 20%, and under 10%, respectively. This is considerably less compared to the persistence rates found by some studies relying on longitudinal survey data [14, 23, 31]. A Danish study including 6630 adolescents with a history of SARS-CoV-2 showed that for many of the symptoms commonly associated with long/post-COVID about half of the patients with PASC reported ongoing symptoms after 6 months and later [14]. In a comparable cohort from the UK, persistence rates after 6 months were in a similar range for typical symptoms such as tiredness or shortness of breath and at about 20% for several other incident symptoms. However, for many of the considered outcomes there were only small changes in symptom persistence after 6–12 months [23]. Another questionnaire-based study from Denmark including 15,041 children and adolescents reported ongoing symptoms in about 40% of the children and about 50% of the adolescents during at least four months after acute COVID-19 [31].

The finding of similar recovery rates in both cohorts in our study does not negate the general burden of disease among the pediatric population caused by post-acute effects of COVID-19. Similar symptom persistence in both groups also means a lasting gap of excess incident health problems in children and adolescents attributed to COVID-19, which results in a long-term impact on pediatric healthcare. The increased incidence of symptoms at more than 6 months post-infection, as was also noted elsewhere [23], further adds to a persisting gap of morbidity among children and adolescents.

Some outcomes, especially cardiac symptoms, gastrointestinal symptoms, fatigue, and inflammatory disorders showed both initial excess risk of onset and prolonged persistence, meaning children and adolescents with a history of COVID-19 are affected more often by these symptoms and take longer to recover from them. In comparison, most neuropsychiatric disorders showed smaller excess risk and almost equal symptom persistence, yet increasing incidence rates in both the COVID-19 and control cohort (Supplementary Fig. 1). This could point to mental health outcomes being raised by social restrictions and closures of childcare or educational infrastructure rather than PASC, which is in line with previous evidence from electronic health records [32].

Many conditions mentioned on the WHO “broad” list of PCC-related symptoms showed IRRs of at least 1.3 in the first quarter of follow-up in the present analysis (e.g. stomachache, headache, fever, cough, dyspnea, diarrhea, nausea, throat/chest pain, palpitations, joint pain, myalgia, mood swings, and dizziness/vertigo) [3]. Of the three most specific PCC symptoms named by the WHO, we can confirm smell/taste disturbance, and fatigue (in terms of ME/CFS and malaise/exhaustion) as relevant health outcomes showing very high IRRs in our data. In contrast, anxiety was not considerably more frequent in the COVID-19 cohort compared to controls (Supplementary Table S3). We therefore suggest reevaluating the PCC-specific symptom list based on accumulating evidence, potentially by including other relevant conditions such as respiratory insufficiency, and inflammatory disorders. Our findings confirm the relevance of cardiovascular symptoms, gastrointestinal symptoms, and fatigue as listed by the Delphi-based PC-COS. By contrast, we found respiratory problems and smell/taste disturbances, which are not included in the PC-COS, as equally important symptoms in children and adolescents based on physician-made diagnoses.

### Strengths and limitations

Major strengths of the present study include the large database of routinely documented healthcare data reflecting the evaluation made by physicians, and the matched control design. Therefore, potential bias due to recall bias or selective self-reporting of symptoms among patients is minimized. Nevertheless, there are several sources of potential bias in secondary analyses of routine healthcare data. First, physicians can only evaluate conditions in children and adolescents seeking medical care. In the COVID-19 cohort, this could have been more often the case, and diagnostic bias due to greater awareness of PASC cannot be excluded. We therefore included two negative outcome controls (prescription glasses, acne) which were consistently found unrelated to COVID-19 in the present analysis. There is however, an unquantifiable risk of underestimating the incidence and persistence of health outcomes, which is likely to affect both study groups equally. One potential reason are barriers in the access to healthcare imposed by the social distancing measures in Germany during the studied period. In addition, the individual burden of the disease, hesitation, or frustration could present other potential barriers for patients to (re-)visit a doctor. In the persistence analysis, we aimed to minimize this bias by allowing for one intermediate “missing” quarter between two recurrent diagnoses to fulfill the persistence criterion. Second, routine healthcare data are not primarily collected for scientific use and we cannot exclude misclassification bias due to uncertainty in the accuracy of diagnoses or diagnosis due to other reasons (e.g. access to further medical care due to the diagnosis of ME/CFS). Third, follow-up in the present study was limited until September 30, 2021. The results of the present study are thus confined to data documented during the earlier phases of the COVID-19 pandemic dominated by the wildtype and alpha variant of SARS-CoV-2.

## Conclusions

Children with COVID-19 had an excess relative risk of 34% to suffer from any of 96 selected symptoms at three months after the index date. Considering altered smell/taste and fatigue as specific conditions of PCC the excess relative risk amounted to 230%. While the relative risk of most common viral symptoms stayed moderately elevated after 12 months of follow-up, there was a noticeable decrease in the risk of PCC-specific symptoms, albeit remaining at a higher overall level. Adolescents were more frequently affected by respiratory problems than children were while inflammatory disorders were more common among children. Tracking diagnoses at the individual level showed that only a small share of pediatric patients suffered from persistent symptoms after one year. In general, these were less than 10% for most symptoms. Among the few conditions that persisted longer following COVID-19 were some less frequent but serious conditions such as ME/CFS, which was especially relevant in adolescents.

## Supplementary Information

Below is the link to the electronic supplementary material.

Supplementary file1 (TIF 3655 KB)

Supplementary file2 (DOCX 891 KB)

## Data Availability

The aggregated data used for the combined analysis of the anonymous individual data from the participating research institutes and health insurance funds can be made available upon request. The data is stored on a secure drive in the ZEGV, to facilitate replication of the results. For assistance in obtaining access to the data, please contact sekretariat.zegv@ukdd.de. The study protocol is available at ClinicalTrials.gov (NCT no. NCT05074953).
