# Supplementary material for: Long/post-COVID in children and adolescents: symptom onset and recovery after one year based on healthcare records in Germany
Source: Infection. 2024 Sep 16;53(1):415–26. doi: 10.1007/s15010-024-02394-8 (PMC11825604; doi:10.1007/s15010-024-02394-8)
Supplement: Supplementary file 2 — Supplementary file2 (DOCX 891 KB) [file 15010_2024_2394_MOESM2_ESM.docx]

Supplementary information

**Manuscript title:** Long/post-COVID in children and adolescents: symptom onset and recovery after one year based on health care records in Germany

# Content

[Content 1](#_Toc169092052)

[A. Analyses by age group 2](#_Toc169092053)

[B. Analyses by sex 4](#_Toc169092054)

[C. Complete list of excess risk of individual health outcomes 6](#_Toc169092055)

[D. Overview of the incidence of studied symptoms and conditions with significant relative risk 8](#_Toc169092056)

[E. Defintion of health outcomes 9](#_Toc169092057)

[F. Definition of health outcome domains 12](#_Toc169092058)

[G. Definition of symptom complexes/groups of diagnoses 12](#_Toc169092059)

[H. Defintion of Covariables 13](#_Toc169092060)

[I. RECORD statement 15](#_Toc169092061)

# Analyses by age group

| **Supplementary table S1** Incidence rate differences (ΔIR) and incidence rate ratios (IRR) of selected symptom complexes (ΔIR > 10/1,000 person-years) in children and adolescents by age group after 0-3 months and after 9-12 months | | | | | | | | |
| --- | --- | --- | --- | --- | --- | --- | --- | --- |
|  |  | **0 to 3 months follow up** | | | **9 to 12 months follow up** | | | |
|  |  | **IR COVID** | **IR Control** | **IRR (95% CI)** | **IR COVID** | **IR Control** | | **IRR (95% CI)** |
| **0-11 years** | **Symptom complexes** |  |  |  |  |  |  |  |
|  | Pulmonary | 49.26 | 26.48 | 1.86 (1.62, 2.13) | 67.80 | 46.29 | 1.46 (1.27, 1.69) | |
|  | Cardiac | 35.83 | 24.23 | 1.48 (1.28, 1.71) | 38.70 | 27.27 | 1.42 (1.18, 1.71) | |
|  | Pain | 50.99 | 32.21 | 1.58 (1.39, 1.80) | 64.07 | 43.38 | 1.48 (1.28, 1.71) | |
|  | Multifactorial | 49.10 | 33.16 | 1.48 (1.30, 1.68) | 62.93 | 44.24 | 1.42 (1.23, 1.65) | |
|  | Gastro-intestinal | 111.45 | 77.37 | 1.44 (1.33, 1.57) | 143.20 | 106.69 | 1.34 (1.22, 1.48) | |
|  | Neuropsychiatric | 112.28 | 86.59 | 1.30 (1.20, 1.41) | 143.20 | 109.10 | 1.31 (1.19, 1.44) | |
|  | Neurological | 175.87 | 147.17 | 1.20 (1.12, 1.27) | 227.48 | 199.23 | 1.14 (1.06, 1.23) | |
| **12-17 years** | **Symptom complexes** |  |  |  |  |  |  |  |
|  | Pulmonary | 45.91 | 16.13 | 2.85 (2.37, 3.42) | 53.11 | 28.43 | 1.87 (1.50, 2.32) | |
|  | Cardiac | 64.89 | 35.57 | 1.82 (1.60, 2.08) | 83.11 | 55.53 | 1.52 (1.24, 1.85) | |
|  | Pain | 119.92 | 75.60 | 1.59 (1.45, 1.74) | 161.86 | 114.33 | 1.42 (1.26, 1.59) | |
|  | Multifactorial | 39.52 | 24.53 | 1.61 (1.37, 1.90) | 56.78 | 37.44 | 1.50 (1.27, 1.76) | |
|  | Gastro-intestinal | 132.39 | 91.77 | 1.44 (1.32, 1.57) | 180.86 | 139.14 | 1.30 (1.17, 1.44) | |
|  | Neuropsychiatric | 169.50 | 123.46 | 1.37 (1.27, 1.48) | 238.56 | 188.03 | 1.27 (1.16, 1.39) | |
|  | Neurological | 112.79 | 81.68 | 1.38 (1.26, 1.51) | 123.40 | 100.79 | 1.22 (1.08, 1.39) | |
| **Total < 18 years** | **Symptom complexes** |  |  |  |  |  |  | |
|  | Pulmonary | 47.79 | 21.93 | 2.18 (1.96, 2.43) | 62.15 | 39.42 | 1.58 (1.40, 1.78) | |
|  | Cardiac | 48.62 | 29.22 | 1.66 (1.51, 1.84) | 55.80 | 38.15 | 1.46 (1.29, 1.65) | |
|  | Pain | 81.32 | 51.30 | 1.59 (1.47, 1.71) | 101.72 | 70.70 | 1.44 (1.31, 1.57) | |
|  | Multifactorial | 44.88 | 29.36 | 1.53 (1.38, 1.69) | 60.56 | 41.62 | 1.46 (1.29, 1.64) | |
|  | Gastro-intestinal | 120.67 | 83.70 | 1.45 (1.37, 1.54) | 157.70 | 119.19 | 1.32 (1.23, 1.42) | |
|  | Neuropsychiatric | 137.46 | 102.82 | 1.34 (1.27, 1.41) | 179.91 | 139.50 | 1.29 (1.21, 1.38) | |
|  | Neurological | 148.11 | 118.35 | 1.25 (1.19, 1.32) | 187.41 | 161.32 | 1.16 (1.09, 1.24) | |

| **Supplementary table S2** Incidence rate differences (ΔIR) and incidence rate ratios (IRR) of overarching health outcome domains in children and adolescents by age group after 0-3 months and after 9-12 months | | | | | | | | | | | | | | | |  |  |
| --- | --- | --- | --- | --- | --- | --- | --- | --- | --- | --- | --- | --- | --- | --- | --- | --- | --- |
|  |  | | **0 to 3 months follow up** | | | | | | **9 to 12 months follow up** | | | | | | |  |  |
|  |  | | **IR COVID** | | **IR Control** | | **IRR (95% CI)** | | **IR COVID** | | **IR Control** | | | **IRR (95% CI)** | |  |  |
| **0-11 years** | **Health outcome domain** | |  |  |  |  |  |  |  |  |  |  |  |  |  |  |  |
|  | Physical | | 288.23 | | 206.59 | | 1.40 (1.33, 1.47) | | 368.67 | | 279.56 | | 1.32 (1.24, 1.40) | | |  |  |
|  | Mental | | 95.14 | | 77.23 | | 1.23 (1.13, 1.34) | | 126.71 | | 97.97 | | 1.29 (1.17, 1.43) | | |  |  |
|  | Overlap | | 238.23 | | 174.69 | | 1.36 (1.29, 1.44) | | 276.93 | | 228.29 | | 1.21 (1.14, 1.30) | | |  |  |
|  | Overall (any symptom) | | 550.84 | | 416.14 | | 1.32 (1.28, 1.37) | | 662.08 | | 538.51 | | 1.23 (1.18, 1.28) | | |  |  |
| **12-17 years** | **Health outcome domain** | |  |  |  |  |  |  |  |  |  |  |  |  |  |  |  |
|  | Physical | | 328.42 | | 222.27 | | 1.48 (1.40, 1.56) | | 416.90 | | 325.45 | | 1.28 (1.20, 1.37) | | |  |  |
|  | Mental | | 134.49 | | 108.31 | | 1.24 (1.14, 1.35) | | 205.59 | | 166.27 | | 1.24 (1.12, 1.36) | | |  |  |
|  | Overlap | | 277.68 | | 180.68 | | 1.54 (1.45, 1.63) | | 354.86 | | 256.64 | | 1.38 (1.28, 1.49) | | |  |  |
|  | Overall (any symptom) | | 671.62 | | 491.72 | | 1.37 (1.32, 1.42) | | 847.77 | | 672.99 | | 1.26 (1.20, 1.32) | | |  |  |
| **Total < 18 years** | **Health outcome domain** | |  | |  | |  | |  | |  | |  | | |  |  |
|  | Physical | | 305.91 | | 213.49 | | 1.43 (1.38, 1.49) | | 387.24 | | 297.23 | | 1.30 (1.25, 1.36) | | |  |  |
|  | Mental | | 112.46 | | 90.91 | | 1.24 (1.17, 1.31) | | 157.08 | | 124.27 | | 1.26 (1.18, 1.36) | | |  |  |
|  | Overlap | | 255.59 | | 177.33 | | 1.44 (1.38, 1.50) | | 306.94 | | 239.21 | | 1.28 (1.22, 1.35) | | |  |  |
|  | Overall (any symptom) | | 603.99 | | 449.40 | | 1.34 (1.31, 1.38) | | 733.58 | | 590.30 | | 1.24 (1.20, 1.28) | | |  |  |
| **Supplementary table S3** Incidence rate differences (ΔIR) and incidence rate ratios (IRR) of symptoms most frequently reported in association with post-COVID condition (PCC) in children and adolescents by age after 0-3 months and after 9-12 months | | | | | | | | | | | | | | | | |  |
|  | |  | | **0 to 3 months follow up** | | | | | | **9 to 12 months follow up** | | | | | | | |
|  | |  | | **IR COVID** | | **IR Control** | | **IRR (95% CI)** | | **IR COVID** | | **IR Control** | | | **IRR (95% CI)** | | |
| **0-11 years** | | **PCC symptoms (WHO)** | |  |  |  |  |  |  |  |  |  |  |  |  |  |  |
|  |  | Smell/taste disturbance | | 3.54 | | 0.40 | | 8.90 (3.47, 22.78) | | 3.58 | | 0.86 | | | 4.16 (1.71, 10.15) | | |
|  |  | Malaise/exhaustion | | 11.51 | | 4.05 | | 2.85 (2.05, 3.95) | | 10.76 | | 5.72 | | | 1.88 (1.28, 2.77) | | |
|  |  | Chronic fatigue syndrome | | 0.41 | | 0.05 | | 7.50 (0.58, 96.58) | | 0.14 | | 0.14 | | | 1.00 (0.06, 15.98) | | |
|  |  | Anxiety disorder | | 9.35 | | 7.60 | | 1.23 (0.93, 1.62) | | 13.95 | | 10.30 | | | 1.35 (1.00, 1.84) | | |
|  |  | PCC overall (including anxiety) | | 24.45 | | 11.90 | | 2.06 (1.68, 2.51) | | 27.95 | | 16.82 | | | 1.66 (1.32, 2.09) | | |
|  |  | PCC overall (without anxiety) | | 15.23 | | 4.50 | | 3.38 (2.50, 4.58) | | 14.19 | | 6.69 | | | 2.12 (1.50, 3.00) | | |
| **12-17 years** | | **PCC symptoms (WHO)** | |  |  |  |  |  |  |  |  |  |  |  |  |  |  |
|  |  | Smell/taste disturbance | | 15.95 | | 1.68 | | 9.50 (5.67, 15.90) | | 9.40 | | 2.45 | | | 3.84 (1.96, 7.54) | | |
|  |  | Malaise/exhaustion | | 33.01 | | 13.58 | | 2.43 (1.98, 2.99) | | 33.09 | | 21.44 | | | 1.54 (1.19, 2.01) | | |
|  |  | Chronic fatigue syndrome | | 2.52 | | 0.37 | | 6.86 (2.23, 21.04) | | 0.46 | | 0.61 | | | 0.75 (0.12, 4.69) | | |
|  |  | Anxiety disorder | | 28.13 | | 21.90 | | 1.28 (1.07, 1.54) | | 50.88 | | 39.86 | | | 1.28 (1.04, 1.56) | | |
|  |  | PCC overall (including anxiety) | | 75.93 | | 36.04 | | 2.11 (1.85, 2.39) | | 89.98 | | 62.31 | | | 1.44 (1.24, 1.69) | | |
|  |  | PCC overall (without anxiety) | | 49.64 | | 15.25 | | 3.25 (2.70, 3.92) | | 41.90 | | 23.85 | | | 1.76 (1.38, 2.23) | | |
| **Total < 18 years** | | **PCC symptoms (WHO)** | |  | |  | |  | |  | |  | | |  | | |
|  |  | Smell/taste disturbance | | 9.00 | | 0.96 | | 9.36 (5.96, 14.7) | | 5.82 | | 1.47 | | | 3.96 (2.31, 6.77) | | |
|  |  | Malaise/exhaustion | | 20.89 | | 8.20 | | 2.55 (2.14, 3.03) | | 19.26 | | 11.71 | | | 1.65 (1.32, 2.05) | | |
|  |  | Chronic fatigue syndrome | | 1.34 | | 0.19 | | 6.96 (2.49, 19.43) | | 0.26 | | 0.32 | | | 0.82 (0.18, 3.76) | | |
|  |  | Anxiety disorder | | 17.49 | | 13.80 | | 1.27 (1.09, 1.48) | | 27.92 | | 21.48 | | | 1.30 (1.10, 1.54) | | |
|  |  | PCC overall (including anxiety) | | 47.11 | | 22.53 | | 2.09 (1.88, 2.33) | | 51.83 | | 34.34 | | | 1.51 (1.33, 1.72) | | |
|  |  | PCC overall (without anxiety) | | 30.38 | | 9.23 | | 3.29 (2.81, 3.85) | | 24.86 | | 13.30 | | | 1.87 (1.53, 2.28) | | |

# Analyses by sex

| **Supplementary table S**4 Incidence rate differences (ΔIR) and incidence rate ratios (IRR) of selected symptom complexes (ΔIR > 10/1,000 person-years) in children and adolescents by sex after 0-3 months and after 9-12 months | | | | | | | | | | | | | | | | |  |
| --- | --- | --- | --- | --- | --- | --- | --- | --- | --- | --- | --- | --- | --- | --- | --- | --- | --- |
|  | |  | | **0 to 3 months follow up** | | | | | | **9 to 12 months follow up** | | | | | | | |
|  | |  | | **IR COVID** | | **IR Control** | | **IRR (95% CI)** | | **IR COVID** | | **IR Control** | | | **IRR (95% CI)** | | |
| **Female** | | **Symptom complexes** | |  |  |  |  |  |  |  |  |  |  |  |  |  |  |
|  |  | Pulmonary | | 46.91 | | 21.55 | | 2.18 (1.86, 2.55) | | 59.41 | | 38.20 | | | 1.56 (1.31, 1.85) | | |
|  |  | Cardiac | | 55.23 | | 35.19 | | 1.57 (1.38, 1.79) | | 61.77 | | 42.05 | | | 1.47 (1.24, 1.74) | | |
|  |  | Pain | | 89.57 | | 58.85 | | 1.52 (1.38, 1.68) | | 111.19 | | 77.65 | | | 1.43 (1.27, 1.62) | | |
|  |  | Multifactorial | | 48.99 | | 32.30 | | 1.52 (1.32, 1.74) | | 66.86 | | 46.75 | | | 1.43 (1.22, 1.68) | | |
|  |  | Gastro-intestinal | | 132.03 | | 94.33 | | 1.40 (1.29, 1.52) | | 164.43 | | 129.67 | | | 1.27 (1.15, 1.40) | | |
|  |  | Neuropsychiatric | | 156.53 | | 114.27 | | 1.37 (1.27, 1.48) | | 199.85 | | 159.57 | | | 1.25 (1.15, 1.37) | | |
|  |  | Neurological | | 138.94 | | 108.79 | | 1.28 (1.18, 1.38) | | 162.97 | | 142.61 | | | 1.14 (1.04, 1.26) | | |
|  | | **Symptom complexes** | |  |  |  |  |  |  |  |  |  |  |  |  |  |  |
| **Male** | | Pulmonary | | 48.62 | | 22.29 | | 2.18 (1.88, 2.54) | | 64.72 | | 40.56 | | | 1.60 (1.36, 1.88) | | |
|  |  | Cardiac | | 42.32 | | 23.55 | | 1.80 (1.54, 2.09) | | 50.17 | | 34.48 | | | 1.46 (1.22, 1.74) | | |
|  |  | Pain | | 73.47 | | 44.12 | | 1.67 (1.49, 1.86) | | 92.80 | | 64.16 | | | 1.45 (1.27, 1.65) | | |
|  |  | Multifactorial | | 40.97 | | 26.56 | | 1.54 (1.33, 1.79) | | 54.62 | | 36.79 | | | 1.48 (1.25, 1.77) | | |
|  |  | Gastro-intestinal | | 109.85 | | 73.58 | | 1.49 (1.37, 1.63) | | 151.36 | | 109.31 | | | 1.38 (1.25, 1.53) | | |
|  |  | Neuropsychiatric | | 119.30 | | 91.91 | | 1.30 (1.20, 1.41) | | 161.12 | | 120.58 | | | 1.34 (1.21, 1.47) | | |
|  |  | Neurological | | 156.85 | | 127.46 | | 1.23 (1.15, 1.32) | | 210.43 | | 178.95 | | | 1.18 (1.08, 1.28) | | |
| **Supplementary table S5** Incidence rate differences (ΔIR) and incidence rate ratios (IRR) of overarching health outcome domains in children and adolescents by sex after 0-3 months and after 9-12 months | | | | | | | | | | | | | | | |  |  |
|  |  | | **0 to 3 months follow up** | | | | | | **9 to 12 months follow up** | | | | | | |  |  |
|  |  | | **IR COVID** | | **IR Control** | | **IRR (95% CI)** | | **IR COVID** | | **IR Control** | | | **IRR (95% CI)** | |  |  |
| **Female** | **Health outcome domain** | |  |  |  |  |  |  |  |  |  |  |  |  |  |  |  |
|  | Physical | | 337.37 | | 238.87 | | 1.41 (1.34, 1.49) | | 412.25 | | 327.51 | | 1.26 (1.18, 1.34) | | |  |  |
|  | Mental | | 128.25 | | 101.35 | | 1.27 (1.17, 1.37) | | 175.33 | | 143.82 | | 1.22 (1.11, 1.34) | | |  |  |
|  | Overlap | | 275.99 | | 191.82 | | 1.44 (1.36, 1.52) | | 321.95 | | 247.58 | | 1.30 (1.21, 1.40) | | |  |  |
|  | Overall (any symptom) | | 656.29 | | 491.85 | | 1.33 (1.29, 1.38) | | 785.61 | | 634.65 | | 1.24 (1.18, 1.29) | | |  |  |
| **Male** | **Health outcome domain** | |  |  |  |  |  |  |  |  |  |  |  |  |  |  |  |
|  | Physical | | 275.97 | | 189.32 | | 1.46 (1.38, 1.54) | | 363.68 | | 268.71 | | 1.35 (1.27, 1.44) | | |  |  |
|  | Mental | | 97.42 | | 80.97 | | 1.20 (1.10, 1.31) | | 139.89 | | 105.86 | | 1.32 (1.19, 1.47) | | |  |  |
|  | Overlap | | 236.17 | | 163.54 | | 1.44 (1.36, 1.53) | | 292.79 | | 231.32 | | 1.27 (1.18, 1.36) | | |  |  |
|  | Overall (any symptom) | | 554.19 | | 408.97 | | 1.36 (1.30, 1.41) | | 684.55 | | 548.52 | | 1.25 (1.19, 1.31) | | |  |  |

| **Supplementary table S6** Incidence rate differences (ΔIR) and incidence rate ratios (IRR) of symptoms most frequently reported in association with post COVID condition in children and adolescents by sex after 0-3 months and after 9-12 months | | | | | | | |  |
| --- | --- | --- | --- | --- | --- | --- | --- | --- |
|  |  | **0 to 3 months follow up** | | | **9 to 12 months follow up** | | | |
|  |  | **IR COVID** | **IR Control** | **IRR (95% CI)** | **IR COVID** | **IR Control** | **IRR (95% CI)** | |
| **Female**  **Male** | **PCC symptoms (WHO)** |  |  |  |  |  |  |  |
|  | Smell/taste disturbance | 11.17 | 1.36 | 8.23 (4.76, 14.24) | 5.82 | 1.67 | 3.49 (1.67, 7.27) | |
|  | Malaise/exhaustion | 25.08 | 10.33 | 2.43 (1.94, 3.04) | 24.02 | 14.31 | 1.68 (1.27, 2.23) | |
|  | Chronic fatigue syndrome | 1.70 | 0.24 | 7.20 (1.92, 27.03) | 0.36 | 0.33 | 1.09 (0.15, 8.09) | |
|  | Anxiety disorder | 23.20 | 18.93 | 1.23 (1.01, 1.48) | 39.14 | 31.10 | 1.26 (1.03, 1.54) | |
|  | PCC overall (including anxiety) | 58.70 | 29.85 | 1.97 (1.72, 2.25) | 66.87 | 46.39 | 1.44 (1.23, 1.69) | |
|  | PCC overall (without anxiety) | 36.77 | 11.75 | 3.13 (2.56, 3.83) | 29.44 | 15.97 | 1.84 (1.42, 2.39) | |
| **Male** | **PCC symptoms (WHO)** |  |  |  |  |  |  |  |
|  | Smell/taste disturbance | 6.94 | 0.59 | 11.85 (5.32, 26.38) | 5.82 | 1.28 | 4.53 (2.06, 9.99) | |
|  | Malaise/exhaustion | 16.93 | 6.19 | 2.73 (2.07, 3.61) | 14.81 | 9.27 | 1.60 (1.13, 2.25) | |
|  | Chronic fatigue syndrome | 0.99 | 0.15 | 6.60 (1.29, 33.65) | 0.17 | 0.31 | 0.55 (0.05, 6.24) | |
|  | Anxiety disorder | 12.12 | 8.97 | 1.35 (1.04, 1.76) | 17.46 | 12.51 | 1.40 (1.03, 1.89) | |
|  | PCC overall (including anxiety) | 36.08 | 15.55 | 2.32 (1.94, 2.77) | 37.66 | 23.00 | 1.64 (1.32, 2.03) | |
|  | PCC overall (without anxiety) | 24.29 | 6.84 | 3.55 (2.75, 4.58) | 20.54 | 10.78 | 1.90 (1.40, 2.58) | |

# Complete list of excess risk of individual health outcomes

| **Supplementary table S7** Incidence rate differences (ΔIR) and incidence rate ratios (IRR) of 96 individual health outcomes and two control endpoints studied in pediatric patients by sex after 0-3 months and after 9-12 months (no values indicate no incident events in COVID-19 and/or control group) | | | | | | | | | | | | | | |  |
| --- | --- | --- | --- | --- | --- | --- | --- | --- | --- | --- | --- | --- | --- | --- | --- |
|  |  | | **0 to 3 months follow up** | | | | | | **9 to 12 months follow up** | | | | | | |
|  |  | | **IR COVID** | | **IR Control** | | **IRR (95% CI)** | | **IR COVID** | | **IR Control** | | **IRR (95% CI)** | | |
| **Individual health outcomes** | |  |  |  |  |  |  |  |  |  |  |  |  |  |  |
| Abdominal pain | | 67.82 | | 45.79 | | 1.48 (1.36, 1.61) | | 75.62 | | 55.06 | | 1.37 (1.23, 1.54) | |  |  |
| Acne | | 32.37 | | 29.29 | | 1.11 (0.99, 1.23) | | 34.16 | | 33.88 | | 1.01 (0.87, 1.17) | |  |  |
| Acute pain | | 1.06 | | 1.14 | | 0.93 (0.53, 1.65) | | 2.12 | | 1.24 | | 1.71 (0.89, 3.31) | |  |  |
| Adjustment disorder | | 26.49 | | 20.19 | | 1.31 (1.16, 1.49) | | 43.20 | | 26.37 | | 1.64 (1.41, 1.90) | |  |  |
| Anuria, oliguria | | 0.05 | | 0.05 | | 1.00 (0.06, 15.99) | | 0.09 | | 0.00 | | - | |  |  |
| Anxiety disorder | | 17.49 | | 13.80 | | 1.27 (1.09, 1.48) | | 27.92 | | 21.48 | | 1.30 (1.10, 1.54) | |  |  |
| Arthritides | | 1.48 | | 1.26 | | 1.18 (0.71, 1.96) | | 0.88 | | 1.40 | | 0.63 (0.29, 1.39) | |  |  |
| Ascites | | 0.09 | | 0.22 | | 0.41 (0.08, 2.15) | | 0.71 | | 0.24 | | 3.00 (0.75, 11.99) | |  |  |
| Behavioral symptoms | | 3.30 | | 3.30 | | 1.00 (0.72, 1.39) | | 5.60 | | 4.08 | | 1.37 (0.94, 2.01) | |  |  |
| Cachexia | | 0.46 | | 0.13 | | 3.53 (0.94, 13.2) | | 0.44 | | 0.29 | | 1.50 (0.38, 6.00) | |  |  |
| Carditis due to viruses | | 0.00 | | 0.00 | | - | | 0.00 | | 0.00 | | - | |  |  |
| Changes in bowel habits | | 0.09 | | 0.06 | | 1.50 (0.17, 13.42) | | 0.09 | | 0.03 | | 3.00 (0.06, 151.14) | |  |  |
| Chest pain | | 0.65 | | 0.22 | | 3.00 (1.05, 8.55) | | 0.18 | | 0.18 | | 1.00 (0.14, 7.10) | |  |  |
| Chronic fatigue syndrome | | 1.34 | | 0.19 | | 6.96 (2.49, 19.43) | | 0.26 | | 0.32 | | 0.82 (0.18, 3.76) | |  |  |
| Cognitive function impairment | | 0.42 | | 0.37 | | 1.12 (0.43, 2.92) | | 0.53 | | 0.47 | | 1.12 (0.35, 3.61) | |  |  |
| Concentration impairment/Concentration deficit | | 0.18 | | 0.27 | | 0.69 (0.19, 2.45) | | 0.18 | | 0.35 | | 0.5 (0.09, 2.73) | |  |  |
| Cough | | 34.38 | | 15.79 | | 2.18 (1.90, 2.49) | | 53.03 | | 33.62 | | 1.58 (1.38, 1.81) | |  |  |
| Covid toe | | 0.05 | | 0.00 | | - | | 0.00 | | 0.03 | | - | |  |  |
| Depression | | 14.55 | | 13.78 | | 1.06 (0.90, 1.24) | | 26.72 | | 21.71 | | 1.23 (1.04, 1.46) | |  |  |
| Developmental delay | | 63.24 | | 58.62 | | 1.08 (0.99, 1.17) | | 100.53 | | 93.19 | | 1.08 (0.98, 1.19) | |  |  |
| Diarrhea | | 48.26 | | 32.32 | | 1.49 (1.34, 1.66) | | 82.11 | | 55.48 | | 1.48 (1.32, 1.66) | |  |  |
| Disorientation | | 0.00 | | 0.00 | | - | | 0.00 | | 0.06 | | - | |  |  |
| Dysgeusia | | 9.00 | | 0.96 | | 9.36 (5.96, 14.7) | | 5.82 | | 1.47 | | 3.96 (2.31, 6.77) | |  |  |
| Dyslexia | | 2.32 | | 2.45 | | 0.95 (0.64, 1.40) | | 2.93 | | 3.01 | | 0.98 (0.60, 1.58) | |  |  |
| Dysmenorrhea | | 30.03 | | 24.23 | | 1.24 (1.10, 1.40) | | 38.60 | | 35.56 | | 1.09 (0.94, 1.25) | |  |  |
| Dysphagia | | 1.62 | | 1.64 | | 0.99 (0.62, 1.58) | | 2.40 | | 2.25 | | 1.07 (0.62, 1.83) | |  |  |
| Dyspnea | | 16.06 | | 6.65 | | 2.42 (1.99, 2.94) | | 13.50 | | 8.40 | | 1.61 (1.24, 2.08) | |  |  |
| Dysuria | | 4.31 | | 2.88 | | 1.50 (1.09, 2.07) | | 3.46 | | 3.18 | | 1.09 (0.69, 1.71) | |  |  |
| Emotional and behavioral disorder | | 44.67 | | 40.30 | | 1.11 (1.01, 1.22) | | 65.63 | | 58.40 | | 1.12 (1.01, 1.26) | |  |  |
| Epistaxis | | 8.79 | | 6.32 | | 1.39 (1.12, 1.74) | | 9.55 | | 7.24 | | 1.32 (0.99, 1.76) | |  |  |
| Eye pain | | 1.39 | | 0.96 | | 1.44 (0.82, 2.52) | | 1.15 | | 1.19 | | 0.96 (0.45, 2.06) | |  |  |
| Facial nerve paralysis | | 0.42 | | 0.26 | | 1.59 (0.56, 4.54) | | 0.53 | | 0.24 | | 2.25 (0.53, 9.52) | |  |  |
| Fever | | 25.57 | | 14.72 | | 1.74 (1.50, 2.01) | | 41.98 | | 23.83 | | 1.76 (1.50, 2.07) | |  |  |
| Flatulence | | 6.52 | | 5.03 | | 1.30 (1.01, 1.67) | | 5.90 | | 4.66 | | 1.27 (0.88, 1.83) | |  |  |
| Gangraen | | 0.09 | | 0.05 | | 2.00 (0.18, 22.05) | | 0.09 | | 0.06 | | 1.5 (0.07, 33.25) | |  |  |
| General symptoms | | 2.18 | | 1.38 | | 1.58 (1.00, 2.49) | | 2.66 | | 1.55 | | 1.71 (0.95, 3.09) | |  |  |
| Hair loss | | 4.73 | | 2.68 | | 1.76 (1.28, 2.44) | | 3.45 | | 2.89 | | 1.19 (0.75, 1.90) | |  |  |
| Headache | | 43.84 | | 25.21 | | 1.74 (1.56, 1.94) | | 59.29 | | 35.20 | | 1.68 (1.48, 1.92) | |  |  |
| Hearing loss/tinnitus | | 8.66 | | 7.36 | | 1.18 (0.95, 1.46) | | 9.92 | | 8.87 | | 1.12 (0.85, 1.47) | |  |  |
| Heart failure | | 0.69 | | 0.22 | | 3.21 (1.14, 9.08) | | 0.62 | | 0.28 | | 2.21 (0.59, 8.33) | |  |  |
| Heart murmurs | | 9.18 | | 6.91 | | 1.33 (1.07, 1.65) | | 9.75 | | 7.52 | | 1.30 (0.97, 1.72) | |  |  |
| Heartburn | | 1.06 | | 0.67 | | 1.59 (0.82, 3.06) | | 1.77 | | 0.54 | | 3.24 (1.31, 8.00) | |  |  |
| Hemorrhage | | 0.23 | | 0.28 | | 0.83 (0.25, 2.73) | | 0.18 | | 0.21 | | 0.86 (0.13, 5.66) | |  |  |
| Hepatomegaly and splenomegaly | | 1.53 | | 0.52 | | 2.96 (1.50, 5.82) | | 1.41 | | 0.96 | | 1.48 (0.68, 3.19) | |  |  |
| Hoarseness | | 2.04 | | 1.32 | | 1.55 (0.97, 2.49) | | 4.09 | | 2.64 | | 1.55 (0.98, 2.46) | |  |  |
| Hyperhidrosis | | 2.13 | | 1.33 | | 1.60 (1.01, 2.56) | | 1.86 | | 1.50 | | 1.23 (0.65, 2.34) | |  |  |
| Hypotension | | 5.08 | | 3.89 | | 1.31 (0.98, 1.74) | | 5.88 | | 6.07 | | 0.97 (0.69, 1.36) | |  |  |
| Impaired balance | | 0.46 | | 0.35 | | 1.30 (0.51, 3.34) | | 0.71 | | 0.41 | | 1.71 (0.55, 5.37) | |  |  |
| Joint pain | | 19.81 | | 14.22 | | 1.39 (1.20, 1.62) | | 24.38 | | 18.44 | | 1.32 (1.10, 1.59) | |  |  |
| Loss of appetite, weight gain/loss, eating disorders | | 11.42 | | 9.25 | | 1.24 (1.02, 1.49) | | 13.49 | | 12.26 | | 1.10 (0.87, 1.39) | |  |  |
| Lymphadenopathy | | 6.21 | | 4.16 | | 1.49 (1.14, 1.95) | | 5.99 | | 4.86 | | 1.23 (0.86, 1.76) | |  |  |
| Malaise/fatigue/exhaustion | | 20.89 | | 8.20 | | 2.55 (2.14, 3.03) | | 19.26 | | 11.71 | | 1.65 (1.32, 2.05) | |  |  |
| Memory impairment | | 0.42 | | 0.58 | | 0.71 (0.30, 1.67) | | 0.71 | | 0.53 | | 1.33 (0.46, 3.84) | |  |  |
| Meningismus | | 0.09 | | 0.02 | | 6.00 (0.15, 234.74) | | 0.26 | | 0.15 | | 1.8 (0.27, 11.95) | |  |  |
| Mood disorder | | 8.24 | | 5.64 | | 1.46 (1.16, 1.84) | | 7.01 | | 5.89 | | 1.19 (0.86, 1.65) | |  |  |
| Movement disorders | | 27.23 | | 21.63 | | 1.26 (1.11, 1.43) | | 42.64 | | 30.77 | | 1.39 (1.20, 1.60) | |  |  |
| Inflammatory disorders | | 1.48 | | 0.35 | | 4.17 (1.90, 9.18) | | 1.77 | | 0.40 | | 4.44 (1.6, 12.35) | |  |  |
| Myalgia | | 19.32 | | 10.22 | | 1.89 (1.60, 2.23) | | 19.83 | | 15.48 | | 1.28 (1.05, 1.57) | |  |  |
| Myocardial infarction | | 0.18 | | 0.12 | | 1.50 (0.32, 7.06) | | 0.35 | | 0.21 | | 1.71 (0.34, 8.61) | |  |  |
| Myocarditis | | 0.00 | | 0.02 | | - | | 0.09 | | 0.16 | | 0.55 (0.05, 6.23) | |  |  |
| Nausea | | 29.62 | | 18.36 | | 1.61 (1.42, 1.84) | | 42.78 | | 31.27 | | 1.37 (1.18, 1.58) | |  |  |
| Neurasthenia | | 2.50 | | 1.37 | | 1.82 (1.16, 2.85) | | 3.18 | | 1.80 | | 1.77 (1.03, 3.05) | |  |  |
| Neurological manifestation of Post-COVID | | 0.09 | | 0.19 | | 0.48 (0.09, 2.59) | | 0.09 | | 0.19 | | 0.46 (0.04, 4.93) | |  |  |
| Obsessive-compulsive disorder | | 1.99 | | 1.66 | | 1.20 (0.77, 1.87) | | 4.42 | | 3.36 | | 1.32 (0.86, 2.01) | |  |  |
| Oedema | | 0.88 | | 0.55 | | 1.58 (0.77, 3.26) | | 1.15 | | 0.99 | | 1.16 (0.52, 2.59) | |  |  |
| Other cardiac arrhythmias | | 4.41 | | 2.80 | | 1.57 (1.14, 2.17) | | 5.25 | | 3.78 | | 1.39 (0.94, 2.06) | |  |  |
| Other coordination disorders/ataxia | | 4.07 | | 3.94 | | 1.03 (0.77, 1.39) | | 4.57 | | 4.41 | | 1.04 (0.70, 1.53) | |  |  |
| Other symptoms of the urinary system | | 1.81 | | 1.37 | | 1.32 (0.82, 2.13) | | 1.95 | | 1.12 | | 1.74 (0.87, 3.47) | |  |  |
| Pain, not elsewhere classified | | 4.99 | | 4.55 | | 1.10 (0.83, 1.44) | | 7.32 | | 5.94 | | 1.23 (0.89, 1.70) | |  |  |
| Paresis | | 0.18 | | 0.15 | | 1.20 (0.28, 5.13) | | 0.26 | | 0.18 | | 1.50 (0.25, 8.97) | |  |  |
| Paresthesia of skin | | 2.27 | | 1.33 | | 1.70 (1.07, 2.69) | | 1.77 | | 1.39 | | 1.28 (0.66, 2.47) | |  |  |
| Pathological findings from male genital tract | | 0.00 | | 0.02 | | - | | 0.00 | | 0.00 | | - | |  |  |
| Pathological lung findings | | 0.23 | | 0.09 | | 2.50 (0.49, 12.88) | | 0.09 | | 0.09 | | 1.00 (0.06, 15.98) | |  |  |
| Pericarditis | | 0.09 | | 0.05 | | 2.00 (0.18, 22.05) | | 0.09 | | 0.12 | | 0.75 (0.06, 10.02) | |  |  |
| Polyuria | | 2.13 | | 1.64 | | 1.30 (0.84, 2.02) | | 1.50 | | 1.81 | | 0.83 (0.44, 1.58) | |  |  |
| Prescription glasses | | 39.87 | | 39.36 | | 1.01 (0.91, 1.13) | | 35.41 | | 33.00 | | 1.07 (0.92, 1.25) | |  |  |
| Pulmonary embolism | | 0.00 | | 0.02 | | - | | 0.00 | | 0.00 | | - | |  |  |
| Rash | | 18.36 | | 13.01 | | 1.41 (1.21, 1.65) | | 17.15 | | 14.63 | | 1.17 (0.95, 1.45) | |  |  |
| Respiratory insufficiency | | 1.67 | | 0.91 | | 1.83 (1.06, 3.17) | | 3.91 | | 1.66 | | 2.36 (1.37, 4.05) | |  |  |
| Seizures | | 4.37 | | 3.02 | | 1.45 (1.05, 1.99) | | 5.31 | | 4.00 | | 1.33 (0.90, 1.96) | |  |  |
| Sensation and perception disorder | | 2.93 | | 1.96 | | 1.49 (1.01, 2.21) | | 3.64 | | 2.83 | | 1.29 (0.81, 2.05) | |  |  |
| Shock | | 0.14 | | 0.27 | | 0.51 (0.13, 2.07) | | 0.53 | | 0.26 | | 2.00 (0.50, 7.99) | |  |  |
| Sinus vein thrombosis | | 0.00 | | 0.00 | | - | | 0.00 | | 0.00 | | - | |  |  |
| Sleep disorders | | 13.67 | | 9.01 | | 1.52 (1.26, 1.82) | | 12.83 | | 8.75 | | 1.47 (1.13, 1.90) | |  |  |
| Somatization disorder | | 20.67 | | 14.40 | | 1.44 (1.24, 1.66) | | 23.59 | | 17.15 | | 1.38 (1.14, 1.66) | |  |  |
| Somnolence, sopor, coma | | 0.28 | | 0.45 | | 0.61 (0.22, 1.68) | | 0.97 | | 0.56 | | 1.74 (0.65, 4.62) | |  |  |
| Speech and language disorders | | 3.53 | | 2.46 | | 1.44 (1.01, 2.04) | | 4.09 | | 3.29 | | 1.24 (0.81, 1.92) | |  |  |
| Stroke | | 0.46 | | 0.31 | | 1.50 (0.56, 4.00) | | 0.62 | | 0.62 | | 1.00 (0.35, 2.85) | |  |  |
| Subcutaneous nodules | | 1.34 | | 1.00 | | 1.34 (0.77, 2.34) | | 1.50 | | 1.09 | | 1.38 (0.66, 2.87) | |  |  |
| Syncope | | 3.86 | | 2.63 | | 1.46 (1.04, 2.05) | | 3.64 | | 3.73 | | 0.98 (0.63, 1.50) | |  |  |
| Tachycardia/Palpitation | | 8.44 | | 4.62 | | 1.83 (1.43, 2.33) | | 8.47 | | 6.05 | | 1.40 (1.03, 1.91) | |  |  |
| Tetany | | 0.00 | | 0.05 | | - | | 0.00 | | 0.00 | | - | |  |  |
| Throat/chest pain | | 24.21 | | 11.93 | | 2.03 (1.74, 2.36) | | 28.59 | | 15.90 | | 1.80 (1.49, 2.17) | |  |  |
| Thrombosis | | 0.32 | | 0.17 | | 1.91 (0.54, 6.75) | | 0.35 | | 0.25 | | 1.41 (0.31, 6.47) | |  |  |
| Urethral discharge | | 0.00 | | 0.02 | | - | | 0.00 | | 0.00 | | - | |  |  |
| Urinary retention | | 0.37 | | 0.19 | | 1.92 (0.59, 6.27) | | 0.44 | | 0.31 | | 1.43 (0.36, 5.60) | |  |  |
| Vertigo | | 11.85 | | 8.18 | | 1.45 (1.19, 1.76) | | 13.59 | | 11.22 | | 1.21 (0.96, 1.53) | |  |  |
| Visual disturbances | | 30.21 | | 27.89 | | 1.08 (0.97, 1.21) | | 31.25 | | 31.00 | | 1.01 (0.86, 1.17) | |  |  |

# Overview of the incidence of studied symptoms and conditions with significant relative risk

**Supplementary Fig. 1** Overview of the incidence of studied symptoms and conditions with significant relative risk

The figure shows symptoms and conditions with significant excess relative risk at three months after acute COVID-19 (i.e. health outcomes with a lower limit of CI 95% greater than 1 in the first and second quarter after index). All symptoms are positioned according to the relative change in incidence rates (IR) over time in the COVID-19 and control group. For each symptom, dot size is proportional to the relative risk (IRR) associated with COVID-19 in the first quarter after index (line) and after 9-12 months (filled), respectively. Different colors are used to denote the frequency (IR) of an onset health condition in the COVID-19 group. To illustrate the changing risk of suffering from symptoms over time, the percentage change of incidence between first and fourth follow-up quarter is tracked in both, COVID-19 and control group, and growth ratios are plotted against each other. Positioning of dots underneath the diagonal line in the plot suggests a decreasing impact of COVID-19 on incident diagnoses i.e. high incidence rates in the COVID-19 group at the start of follow-up are countered by an increasing incidence in the control group. Symptoms along the diagonal line show a similar growth ratio suggesting a near constant impact of COVID-19 over time. Conditions towards the top left indicate an increasing relative risk in the COVID-19 group as follow-up time progresses. Note: Due to very small number of diagnoses for Chronic fatigue (ME/CFS) in the COVID-19 and control group we could not calculate the point estimate after 9-12 months. In this circumstance, the IRR shown refers to 6-9 months (graphic created using the ggplot2 package in R).


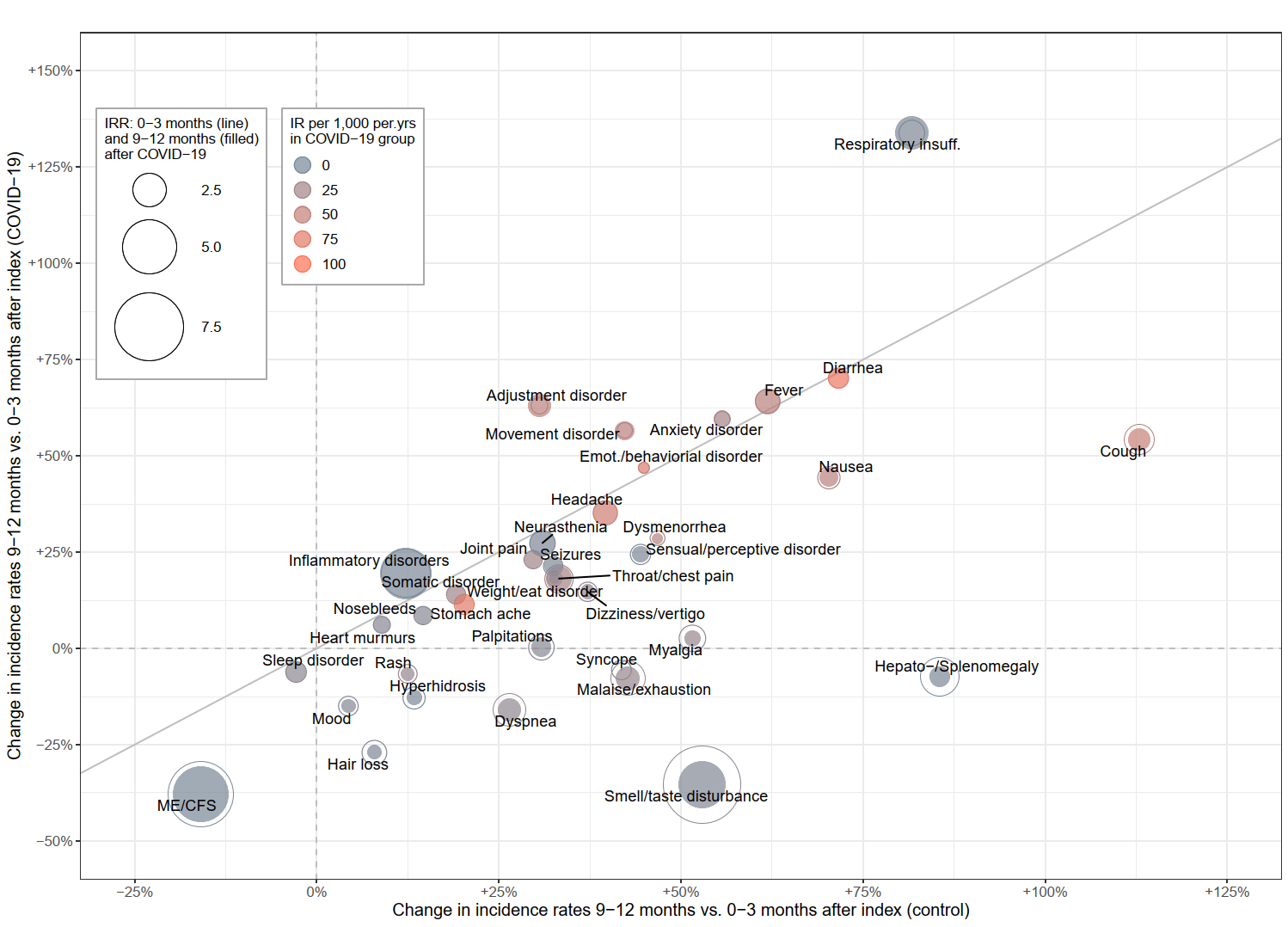


# Defintion of health outcomes

**Supplementary table S8** Definition of incident health outcomes

| **Health outcome** | **ICD-10 (German modification) codes**  Any incident inpatient or outpatient diagnosis during observational period (post-index) |
| --- | --- |
| Abdominal pain | R10.1 R10.2 R10.3 R10.4 |
| Acne | L70.0 L70.1 L70.2 L70.3 L70.4 L70.5 L70.8 L70.9 |
| Acute pain | R52.0 |
| Adjustment disorder | F43.0 F43.1 F43.2 F43.8 F43.9 |
| Anuria, oliguria | R34 |
| Anxiety disorder | F40.0 F40.00 F40.01 F40.1 F40.2 F40.8 F40.9 F41.0 F41.1 F41.2 F41.3 F41.8 F41.9 |
| Arthritides | M02.0 M02.00 M02.01 M02.02 M02.03 M02.04 M02.05 M02.06 M02.07 M02.08 M02.09 M02.1 M02.10 M02.11 M02.12 M02.13 M02.14 M02.15 M02.16 M02.17 M02.18 M02.19 M02.2 M02.20 M02.21 M02.22 M02.23 M02.24 M02.25 M02.26 M02.27 M02.28 M02.29 M02.3 M02.30 M02.31 M02.32 M02.33 M02.34 M02.35 M02.36 M02.37 M02.38 M02.39 M02.8 M02.80 M02.81 M02.82 M02.83 M02.84 M02.85 M02.86 M02.87 M02.88 M02.89 M02.9 M02.90 M02.91 M02.92 M02.93 M02.94 M02.95 M02.96 M02.97 M02.98 M02.99 M13.0 M13.1 M13.11 M13.12 M13.13 M13.14 M13.15 M13.16 M13.17 M13.18 M13.19 M13.8 M13.80 M13.81 M13.82 M13.83 M13.84 M13.85 M13.86 M13.87 M13.88 M13.89 M13.9 M13.90 M13.91 M13.92 M13.93 M13.94 M13.95 M13.96 M13.97 M13.98 M13.99 |
| Ascites | R18 |
| Behavioral symptoms | R46.0 R46.1 R46.2 R46.3 R46.4 R46.5 R46.6 R46.7 R46.8 |
| Cachexia | R64 |
| Carditis due to viruses | B33.2 |
| Changes in bowel habits | R19.4 |
| Chest pain | R07.1 |
| Chronic fatigue syndrome | G93.3 |
| Cognitive function impairment | F06.7 U51.0 U51.00 U51.01 U51.02 U51.1 U51.10 U51.11 U51.12 U51.2 U51.20 U51.21 U51.22 |
| Concentration impairment/deficit | R41.8 |
| Cough | R05 |
| Covid toe | L93.2 |
| Depression | F32.0 F32.1 F32.2 F32.3 F32.8 F32.9 F33.0 F33.1 F33.2 F33.3 F33.4 F33.8 F33.9 F34.1 |
| Developmental delay | F80.0 F80.1 F80.2 F80.20 F80.28 F80.3 F80.8 F80.9 F81.0 F81.1 F81.2 F81.3 F81.8 F81.9 F82.0 F82.1 F82.2 F82.9 F83 F84.0 F84.1 F84.2 F84.3 F84.4 F84.5 F84.8 F84.9 F88 F89 |
| Diarrhea | A09.0 A09.9 A58 K52.9 R19.4 R19.5 |
| Disorientation | R41.0 |
| Disturbances of smell and taste | R43.0 R43.2 R43.8 |
| Dyslexia | R48.0 R48.1 R48.2 R48.8 |
| Dysmenorrhea | N91.0 N91.1 N91.2 N91.3 N91.4 N91.5 N92.0 N92.1 N92.2 N92.3 N92.4 N92.5 N92.6 N94.0 N94.1 N94.2 N94.3 N94.4 N94.5 N94.6 N94.8 N94.9 |
| Dysphagia | R13.0 R13.1 R13.9 |
| Dyspnea | R06.0 R06.2 R06.88 |
| Dysuria | R30.0 R30.1 R30.9 |
| Emotional and behavioral disorder | F54 F59 F91.0 F91.1 F91.2 F91.3 F91.8 F91.9 F92.0 F92.8 F92.9 F93.0 F93.1 F93.2 F93.3 F93.8 F93.9 F95.0 F95.1 F95.2 F95.8 F95.9 F98.8 F98.9 |
| Epistaxis | R04.0 |
| Eye pain | H57.1 |
| Facial nerve paralysis | G51.0 G51.1 G51.2 G51.3 G51.4 G51.8 G51.9 |
| Fever | R50.2 R50.8 R50.80 R50.88 R50.9 |
| Flatulence | R14 |
| Gangraen | R02.0 R02.00 R02.01 R02.02 R02.03 R02.04 R02.05 R02.06 R02.07 R02.09 R02.8 |
| General symptoms | R68.0 R68.1 R68.2 R68.3 R68.8 |
| Hair loss | L63.9 L65.8 L65.9 |
| Headache | R51 |
| Hearing loss/tinnitus | H91.9 H93.1 H93.2 |
| Heart failure | I11.0 I13.0 I13.2 I50.0 I50.00 I50.01 I50.02 I50.03 I50.04 I50.05 I50.1 I50.11 I50.12 I50.13 I50.14 I50.19 I50.9 |
| Heart murmurs | R01.0 R01.1 R01.2 |
| Heartburn | R12 |
| Hemorrhage | R58 |
| Hepatomegaly and splenomegaly | R16.0 R16.1 R16.2 |
| Hoarseness | R49.0 R49.1 R49.2 R49.8 |
| Hyperhidrosis | R61.0 R61.1 R61.9 |
| Hypotension | I95.0 I95.1 I95.8 I95.9 |
| Impaired balance | R29.6 |
| Joint pain | M25.4 M25.5 M25.8 M25.9 |
| Loss of appetite, weight gain/loss, eating disorders | F50.0 F50.00 F50.01 F50.08 F50.1 F50.2 F50.3 F50.4 F50.5 F50.8 F50.9 R63.0 R63.4 R63.5 |
| Lymphadenopathy | R59.0 R59.1 R59.9 |
| Malaise/fatigue/exhaustion | R53. |
| Memory impairment | R41.0 R41.1 R41.2 R41.3 R41.8 |
| Meningismus | R29.1 |
| Mood disorder | R45.0 R45.1 R45.2 R45.3 R45.4 R45.5 R45.6 R45.7 R45.8 |
| Movement disorders | F90.0 F90.1 F90.8 F90.9 G25.0 G25.2 G25.5 G25.8 R25.0 R25.1 R25.2 R25.3 R25.8 R26.0 R26.1 R26.2 R26.3 R26.8 |
| Multisystemic inflammatory syndrome | D76.4 M08.2 M30.3 R65.0 R65.1 R65.2 R65.3 R65.9 U10.9 |
| Myalgia | M79.1 M79.2 M79.6 |
| Myocardial infarction | I20.0 I20.1 I20.8 I20.9 I21.0 I21.1 I21.2 I21.3 I21.4 I21.9 I22.0 I22.1 I22.8 I22.9 I23.0 I23.1 I23.2 I23.3 I23.4 I23.5 I23.6 I23.8 I24.0 I24.1 I24.8 I24.9 I25.0 I25.1 I25.10 I25.11 I25.12 I25.13 I25.14 I25.15 I25.16 I25.19 I25.2 I25.20 I25.21 I25.22 I25.29 I25.3 I25.4 I25.5 I25.6 I25.8 I25.9 |
| Myocarditis | I40.0 I40.1 I40.8 I40.9 |
| Nausea | R11. |
| Neurasthenia | F48.0 |
| Neurological manifestation of Post-COVID | G57.3 G61.0 G72.80 |
| Obsessive-compulsive disorder | F42.0 F42.1 F42.2 F42.8 F42.9 |
| Oedema | R60.0 R60.1 R60.9 |
| Other cardiac arrhythmias | I44.0 I44.1 I44.2 I44.3 I44.4 I44.5 I44.6 I44.7 I45.0 I45.1 I45.2 I45.3 I45.4 I45.5 I45.6 I45.8 I45.9 I46.0 I46.1 I46.9 I47.0 I47.1 I47.2 I47.9 I48.0 I48.1 I48.2 I48.3 I48.4 I48.9 I49.1 I49.2 I49.3 I49.5 I49.8 I49.9 |
| Other coordination disorders/ataxia | R27.0 R27.8 |
| Other symptoms of the urinary system | R39.0 R39.1 R39.2 R39.8 |
| Pain, not elsewhere classified | R52.0 R52.1 R52.2 R52.9 |
| Paresis | G82.00 G82.02 G82.35 G83.0 G83.1 G83.2 G83.3 G83.6 G83.8 G83.9 |
| Paresthesia of skin | R20.0 R20.1 R20.2 R20.3 R20.8 |
| Pathological findings from male genital tract | R86.0 R86.1 R86.2 R86.3 R86.4 R86.5 R86.6 R86.7 R86.8 R86.9 |
| Pathological lung findings | J84.0 J84.1 J93.8 J98.4 |
| Pathological reflexes | R29.2 |
| Pericarditis | I30.0 I30.8 I30.9 I31.9 |
| Polyuria | R35 |
| Pulmonary embolism | I26.0 I26.9 |
| Rash | R21 R23.0 R23.1 R23.2 R23.3 R23.4 R23.8 |
| Respiratory insufficiency | J96.0 J96.00 J96.01 J96.09 J96.1 J96.10 J96.11 J96.19 J96.9 J96.90 J96.91 J96.99 |
| Seizures | G40.0 G40.00 G40.01 G40.02 G40.08 G40.09 G40.1 G40.2 G40.3 G40.4 G40.5 G40.6 G40.7 G40.8 G40.9 R56.0 R56.8 |
| Sensation and perception disorder | R44.0 R44.1 R44.2 R44.3 R44.8 |
| Shock | R57.0 R57.1 R57.2 R57.8 R57.9 |
| Sinus vein thrombosis | I67.8 |
| Sleep disorders | F51 G47.0 G47.1 G47.2 G47.8 G47.9 |
| Somatization disorder | F45.0 F45.1 F45.2 F45.3 F45.30 F45.31 F45.32 F45.33 F45.34 F45.37 F45.38 F45.39 F45.4 F45.40 F45.41 F45.8 F45.9 |
| Somnolence, sopor, coma | R40.0 R40.1 R40.2 |
| Speech and language disorders | R47.0 R47.1 R47.8 |
| Stroke | I60.0 I60.1 I60.2 I60.3 I60.4 I60.5 I60.6 I60.7 I60.8 I60.9 I61.0 I61.1 I61.2 I61.3 I61.4 I61.5 I61.6 I61.8 I61.9 I62.0 I62.00 I62.01 I62.02 I62.09 I62.1 I62.9 I63.0 I63.1 I63.2 I63.3 I63.4 I63.5 I63.6 I63.8 I63.9 I64 I65.0 I65.1 I65.2 I65.3 I65.8 I65.9 I66.0 I66.1 I66.2 I66.3 I66.4 I66.8 I66.9 I67.0 I67.1 I67.10 I67.11 I67.2 I67.3 I67.4 I67.5 I67.6 I67.7 I67.8 I67.80 I67.88 I67.9 I68.0 I68.1 I68.2 I68.8 I69.0 I69.1 I69.2 I69.3 I69.4 I69.8 |
| Subcutaneous nodules | R22.0 R22.1 R22.2 R22.3 R22.4 R22.7 R22.9 |
| Syncope | R55 |
| Tachycardia/Palpitation | I49.0 I49.4 R00.0 R00.1 R00.2 R00.3 R00.8 |
| Tetany | R29.0 |
| Throat/chest pain | R07.0 R07.1 R07.2 R07.3 R07.4 R09.1 |
| Thrombosis | I80.0 I80.1 I80.2 I80.20 I80.28 I80.3 I80.8 I80.80 I80.81 I80.88 I80.9 |
| Urethral discharge | R36 |
| Urinary retention | R33 |
| Vertigo | H81.0 H81.1 H81.2 H81.3 H81.4 H81.8 H81.9 H82 R42 |
| Visual disturbances | H53.0 H53.1 H53.2 H53.3 H53.4 H53.5 H53.6 H53.8 H53.9 |

# Definition of health outcome domains

**Supplementary table S9** Definition of overarching domains of incident health outcomes

| **Health outcome domain** | **Health outcomes**  At least one incident health outcome in the observational period |
| --- | --- |
| Mental | Adjustment disorder; Anxiety disorder; Behavioral symptoms; Cognitive function impairment; Concentration impairment/Concentration deficit; Depression; Disorientation; Emotional and behavioral disorder; Mood disorder; Obsessive‐compulsive disorder; Somatization disorder |
| Overlap (physical/mental) | Abdominal pain; Acute pain; Cachexia; Changes in bowel habits; Chronic fatigue syndrome; Developmental delay; Dysuria; Eye pain; General symptoms; Headache; Hyperhidrosis; Joint pain; Loss of appetite, weight gain/loss, eating disorders; Malaise/fatigue/exhaustion; Memory impairment; Myalgia; Neurasthenia; Other coordination disorders/ataxia; Pain, not elsewhere classified; Paresthesia of skin; Post‐COVID; Sensation and perception disorder; Sleep disorders; Somnolence, sopor, coma; Throat/chest pain |
| Physical | Anuria, oliguria; Arthritides; Ascites; Carditis due to viruses; Cough; Covid toe; Diarrhea; Disturbances of smell and taste; Dyslexia; Dysmenorrhea; Dysphagia; Dyspnea; Epistaxis; Facial nerve paralysis; Fever; Flatulence; Gangraen; Hair loss; Hearing loss/tinnitus; Heart failure; Heart murmurs; Heartburn; Hemorrhage; Hepatomegaly and splenomegaly; Hoarseness; Hypotension; Impaired balance; Lymphadenopathy; Meningismus; Movement disorders; Multisystemic inflammatory syndrome; Myocardial infarction; Myocarditis; Nausea; Neurological manifestation of Post‐COVID; Oedema; Other cardiac arrhythmias; Other symptoms of the urinary system; Paresis; Pathological findings from male genital tract; Pathological lung findings; Pathological reflexes; Pericarditis; Polyuria; Pulmonary embolism; Rash; Respiratory insufficiency; Seizures; Shock; Sinus vein thrombosis; Speech and language disorders; Stroke; Subcutaneous nodules; Syncope; Tachycardia/Palpitation; Tetany; Thrombosis; Urethral discharge; Urinary retention; Vertigo; Visual disturbances |

# Definition of symptom complexes/groups of diagnoses

**Supplementary table S10** Definition of symptom complexes/diagnostic groups of incident health outcomes

| **Symptom complex/group of diagnoses** | **Health outcomes** |
| --- | --- |
| Cardiac | Carditis due to viruses; Heart failure; Heart murmurs; Hypotension; Myocardial infarction; Myocarditis; Other cardiac arrhythmias; Pericarditis; Shock; Syncope; Tachycardia/Palpitation; Throat/chest pain |
| Dermatological | Hair loss; Rash; Subcutaneous nodules |
| ENT | Hearing loss/tinnitus; Hoarseness |
| Gastrointestinal | Abdominal pain; Ascites; Changes in bowel habits; Diarrhea; Flatulence; Heartburn; Hepatomegaly and splenomegaly; Nausea |
| Gynecological/urogenital | Dysmenorrhea; Pathological findings from male genital tract; Urethral discharge |
| Multifactorial | Cachexia; Fever; General symptoms; Hyperhidrosis; Loss of appetite, weight gain/loss, or eating disorders; Lymphadenopathy; Oedema |
| Locomotor system | Arthritides |
| Nephrological | Anuria, oliguria; Dysuria; Other symptoms of the urinary system; Polyuria; Urinary retention |
| Neurological | Abnorm reflexes; Developmental delay; Dysgeusia; Dyslexia; Dysphagia; Facial nerve paralysis; Impaired balance; Meningismus; Movement disorders; Neurological manifestation of Post-COVID; Paresis; Paresthesia of skin; Seizures; Sensation and perception disorder; Sinus vein thrombosis; Sleep disorders; Speech and language disorders; Stroke; Tetany; Vertigo; Visual disturbances |
| Neuropsychiatric | Adjustment disorder; Anxiety disorder; Other coordination disorders/ataxia; Cognitive function impairment; Somnolence, sopor, coma; Concentration impairment/Concentration deficit; Depression; Disorientation; Emotional and behavioral disorder; Chronic fatigue syndrome; Memory impairment; Mood disorder; Neurasthenia; Obsessive-compulsive disorder; Somatization disorder; Behavioral symptoms; Malaise/fatigue/exhaustion |
| Pain | Acute pain;  Eye pain; Headache; Joint pain; Myalgia; Pain, not elsewhere classified |
| Pulmonary | Cough; Dyspnea; Pathological lung findings; Pulmonary embolism; Respiratory insufficiency |
| Coagulation disorder | Covid toe; Epistaxis; Gangraen; Hemorrhage; Thrombosis |

# Defintion of Covariables

**Supplementary table S11** Definition of covariables (general characteristics)

| **Covariable** | **Defintion** |
| --- | --- |
| Age | Age on 2020-12-31 |
| Sex | Sex on 2020-12-31 |
| Inpatient health care utilization | At least one hospital stay in the four quarters preceding the index |
| Outpatient health care utilization | Number of quarters with documented outpatient health services in the four quarters preceding the index |

**Supplementary table S12** Definition of covariables (comorbidities)

| **Covariable** | **ICD-10 (German modification) codes** At least one inpatient diagnosis or two outpatient diagnoses in the four quarters preceding the index |
| --- | --- |
| Asthma with drug therapy | ICD-10: J45.9, J45.8, J45.1, J45.0 |
| Other Diabetes with insulin | ICD-10: E11.0, E11.01, E11.1, E11.11, E11.2, E11.20, E11.21, E11.3, E11.30, E11.31, E11.4, E11.40, E11.41, E11.5, E11.50, E11.51, E11.6, E11.60, E11.61, E11.7, E11.72, E11.73, E11.74, E11.75, E11.8, E11.80, E11.81, E11.9, E11.90, E11.91, E12.0, E12.01, E12.1, E12.11, E12.2, E12.20, E12.21, E12.3, E12.30, E12.31, E12.4, E12.40, E12.41, E12.5, E12.50, E12.51, E12.6, E12.60, E12.61, E12.7, E12.72, E12.73, E12.74, E12.75, E12.8, E12.80, E12.81, E12.9, E12.90, E12.91, E13.0, E13.01, E13.1, E13.11, E13.2, E13.20, E13.21, E13.3, E13.30, E13.31, E13.4, E13.40, E13.41, E13.5, E13.50, E13.51, E13.6, E13.60, E13.61, E13.7, E13.72, E13.73, E13.74, E13.75, E13.8, E13.80, E13.81, E13.9, E13.90, E13.91, E14.0, E14.01, E14.1, E14.11, E14.2, E14.20, E14.21, E14.3, E14.30, E14.31, E14.4, E14.40, E14.41, E14.5, E14.50, E14.51, E14.6, E14.60, E14.61, E14.7, E14.72, E14.73, E14.74, E14.75, E14.8, E14.80, E14.81, E14.9, E14.90, E14.91 |
| Down syndrome | ICD-10: Q90.9, Q90.0, Q90.1, Q90.2 |
| Cancer | ICD-10: C91.5, C91.51, C91.50, C94.0, C94.01, C94.00, C95.0, C95.01, C95.00, C91.0, C91.01, C91.00, C94.2, C94.21, C94.20, C93.0, C93.01, C93.00, C92.0, C92.01, C92.00, C92.6, C92.61, C92.60, C92.8, C92.81, C92.80, C92.5, C92.51, C92.50, C94.4, C94.41, C94.40, C92.4, C92.41, C92.40, C84.7, C84.6, C86.5, C92.2, C92.21, C92.20, C94.8, C86.4, C88.9, C88.91, C88.90, C96.9, C96.2, C83.7, C85.1, C95.1, C95.11, C95.10, C91.1, C91.11, C91.10, C92.1, C92.11, C92.10, C93.1, C93.11, C93.10, C82.5, C83.3, C90.2, C90.21, C90.20, C88.4, C88.41, C88.40, C86.0, C82.0, C82.1, C82.2, C82.3, C82.4, C82.9, C81.2, C91.4, C91.41, C91.40, C86.1, C96.8, C81.9, C88.3, C88.31, C88.30, C93.3, C93.31, C93.30, C83.0, C82.6, C84.8, C95.9, C95.91, C95.90, C95.8, C91.9, C91.91, C91.90, C83.5, C81.3, C81.4, C88.0, C88.01, C88.00, C83.1, C94.3, C94.31, C94.30, C85.2, C93.9, C93.91, C93.90, C96.0, C96.5, C90.0, C90.01, C90.00, C84.0, C94.6, C94.61, C94.60, C92.9, C92.91, C92.90, C92.3, C92.31, C92.30, C83.9, C81.0, C81.1, C85.9, C84.4, C90.1, C90.11, C90.10, C86.6, C91.3, C91.31, C91.30, C91.6, C91.61, C91.60, C91.8, C91.81, C91.80, C84.9, C96.4, C84.1, C90.3, C90.31, C90.30, C88.7, C88.71, C88.70, C95.7, C95.71, C95.70, C91.7, C91.71, C91.70, C93.7, C93.71, C93.70, C92.7, C92.71, C92.70, C96.7, C94.7, C94.71, C94.70, C85.7, C83.8, C84.5, C88.2, C88.21, C88.20, C81.7, C82.7, C86.3, C86.2, C96.6, C79.81, C79.82, C79.1, C79.2, C78.7, C78.0, C79.7, C79.0, C78.2, C79.86, C79.85, C78.5, C78.4, C79.3, C79.5, C78.1, C79.6, C79.83, C78.6, C79.9, C79.8, C79.88, C78.3, C79.4, C78.8, C77.3, C77.4, C77.2, C77.5, C77.1, C77.0, C77.8, C77.9, C79.84, C22.3, C19, C23, C64, C07, C58, C61, C73, C33, C52, C51.2, C51.0, C51.1, C41.4, C41.32, C41.0, C41.01, C40.1, C40.3, C40.2, C41.02, C41.30, C41.3, C40.0, C41.31, C41.1, C41.2, C65, C56, C12, C20, C37, C66, C55, C01, C15.2, C24.1, C21.1, C16.3, C21.0, C67.1, C18.1, C13.1, C39.8, C69.8, C69.9, C00.2, C00.0, C00.1, C75.8, C49.1, C49.2, C49.4, C49.5, C49.0, C49.6, C49.3, C49.8, C49.9, C34.9, C34.8, C50.8, C50.9, C50.0, C72.1, C53.8, C53.9, C69.3, C18.2, C18.6, C18.7, C18.4, C54.8, C54.9, C16.2, C62.1, C75.2, C25.3, C17.8, C17.9, C17.0, C62.0, C53.1, C75.9, C25.4, C54.1, C53.0, C75.3, C24.0, C18.3, C18.5, C09.0, C71.1, C54.3, C16.1, C24.8, C24.9, C05.8, C05.9, C09.1, C72.8, C71.8, C71.9, C08.1, C08.0, C60.1, C75.5, C75.4, C32.0, C16.6, C08.9, C08.8, C67.8, C67.9, C67.5, C68.9, C68.8, C05.0, C34.0, C38.0, C38.8, C67.4, C38.2, C13.2, C11.1, C10.3, C70.0, C71.7, C71.5, C62.9, C13.8, C13.9, C75.1, C17.2, C26.0, C54.0, C17.1, C16.0, C10.4, C16.5, C21.2, C40.8, C40.9, C41.8, C41.9, C18.8, C18.9, C69.0, C69.1, C32.8, C32.9, C32.3, C67.2, C22.9, C57.1, C57.2, C00.8, C14.8, C00.9, C00.5, C00.6, C14.2, C16.8, C16.9, C63.8, C63.9, C17.3, C38.3, C70.9, C26.1, C34.2, C30.1, C06.9, C04.8, C04.9, C54.2, C72.3, C72.4, C48.1, C30.0, C31.9, C31.8, C11.8, C11.9, C63.0, C74.9, C74.1, C74.0, C75.0, C72.2, C39.0, C11.0, C50.4, C50.2, C03.0, C34.1, C00.3, C71.4, C69.6, C10.8, C10.9, C15.8, C15.4, C15.9, C15.3, C15.5, C67.6, C25.8, C25.9, C25.0, C25.1, C25.2, C57.3, C68.1, C71.3, C60.8, C60.9, C60.2, C47.1, C47.2, C47.4, C47.5, C47.0, C47.6, C47.3, C47.8, C47.9, C48.2, C14.0, C38.4, C60.0, C16.4, C50.6, C13.0, C21.8, C69.2, C06.2, C48.0, C48.8, C72.0, C70.1, C63.1, C11.2, C10.2, C04.1, C31.1, C31.2, C31.0, C31.3, C63.2, C63.7, C57.7, C25.7, C72.5, C06.8, C32.2, C32.1, C71.2, C15.1, C09.8, C09.9, C69.5, C67.0, C57.0, C39.9, C26.9, C50.5, C50.3, C03.1, C34.3, C00.4, C67.7, C68.0, C57.4, C05.2, C10.0, C26.8, C06.1, C67.3, C02.3, C04.0, C38.1, C10.1, C11.3, C51.8, C51.9, C06.0, C57.8, C57.9, C05.1, C03.9, C18.0, C50.1, C72.9, C71.6, C71.0, C15.0, C69.4, C02.8, C02.9, C02.1, C02.0, C02.4, C02.2, C43.4, C43.8, C43.9, C43.0, C43.6, C43.7, C43.1, C43.2, C43.5, C43.3, C22.2, C22.1, C46.0, C46.3, C46.2, C46.1, C46.8, C46.7, C46.9, C22.0, C45.0, C45.2, C45.1, C45.7, C45.9, C22.7, C22.4 |
| Immuno-compromising diseases | ICD-10: D57.0, D57.1, D57.2, D57.3, D57.8, D68.0, D68.00, D68.01, D68.09, D68.1, D68.2, D68.20, D68.21, D68.22, D68.23, D68.24, D68.25, D68.26, D68.28, D68.3, D68.31, D68.32, D68.33, D68.34, D68.35, D68.38, D68.4, D68.5, D68.6, D68.8, D68.9, D70.0, D70.1, D70.10, D70.11, D70.12, D70.13, D70.14, D70.18, D70.19, D70.3, D70.5, D70.6, D70.7, D71, D72.0, D72.1, D72.8, D72.9, D73.0, D76.1, D76.2, D76.3, D76.4, D83.0, D83.1, D83.2, D83.8, D83.9, D84.0, D84.1, D84.8, D84.9, D89.0, D89.1, D89.2, D89.3, D89.8, D89.9, D90, I88.0, I88.1, I88.8, I88.9, M35.9 |
| Immunosuppressive therapy | ATC: L04, H02AB, H02B |
| Immune blood disease | ICD-10: D81.3, D80.6, D82.1, D81.6, D81.7, D80.0, D82.4, D80.5, D82.9, D82.2, D82.3, D80.9, D82.8, D81.9, D81.4, D80.1, D81.5, D81.2, D81.1, D81.0, D80.2, D80.4, D80.3, D80.8, D81.8, D77, D80.7, D82.0 |
| Obesity | ICD-10: E66.01, E66.02, E66.05, E66.91, E66.92, E66.95, E66.11, E66.12, E66.15, E66.81, E66.82, E66.85, E66.21, E66.22, E66.25 |
| Epilepsy | ICD-10: G40.0, G40.00, G40.01, G40.02, G40.08, G40.09, G40.1, G40.2, G40.3, G40.4, G40.5, G40.6, G40.7, G40.8, G40.9, G41.0, G41.1, G41.2, G41.8, G41.9 |
| Psychomotor disorder | ICD-10: F82.0, F82.1, F82.2, F82.9 |
| Bronchopulmonary Dysplasia | ICD-10: P27.1 |
| Heart defects | ICD-10: Q20.0, Q20.1, Q20.2, Q20.3, Q20.4, Q20.5, Q20.6, Q20.8, Q20.9, Q21.0, Q21.1, Q21.2, Q21.3, Q21.4, Q21.8, Q21.80, Q21.88, Q21.9, Q22.0, Q22.1, Q22.2, Q22.3, Q22.4, Q22.5, Q22.6, Q22.8, Q22.9, Q23.0, Q23.1, Q23.2, Q23.3, Q23.4, Q23.8, Q23.9, Q24.0, Q24.1, Q24.2, Q24.3, Q24.4, Q24.5, Q24.6, Q248, Q249, Q25.0, Q25.1, Q25.2, Q25.3, Q25.4, Q25.5, Q25.6, Q25.7, Q258, Q259, Q26.0, Q26.1, Q26.2, Q26.3, Q26.4, Q26.5, Q26.6, Q26.8, Q26.9, Q27.0, Q27.1, Q27.2, Q27.3, Q27.4, Q278, Q279, Q28.0, Q28.00, Q28.01, Q28.08, Q28.09, Q28.1, Q28.10, Q28.11, Q28.18, Q28.19, Q28.2, Q28.20, Q28.21, Q28.28, Q28.29, Q28.3, Q28.30, Q28.31, Q28.38, Q28.39, Q28.8, Q28.80, Q28.81, Q28.88, Q28.9 |
| Autoimmune disorders  (including atopic dermatitis) | ICD-10: L63.0, L63.1, L63.8, L63.9, L13.0, L20.0, L20.8, L20.9, L93.0, L93.1, L93.2, L94.0, L12.0, L12.1, L10.0, L10.1, L10.2, L10.3, L10.4, L40.0, L40.1, L40.2, L40.3, L40.4, L40.5, L40.70, L40.8, L40.9, M35.0, L80, E27.1, E05.0, E05.1, E05.2, E05.3, E05.4, E05.5, E05.8, E05.9, E06.2, E10.0, E10.01, E10.1, E10.11, E10.2, E10.20, E10.21, E10.3, E10.30, E10.31, E10.4, E10.40, E10.41, E10.5, E10.50, E10.51, E10.6, E10.60, E10.61, E10.7, E10.72, E10.73, E10.74, E10.75, E10.8, E10.80, E10.81, E10.9, E10.90, E10.91, O24.0, E06.3, K50.0, K50.1, K50.8, K50.80, K50.81, K50.82, K50.88, K50.9, K75.4, K51.0, K51.2, K51.3, K51.4, K51.5, K51.8, K51.9, K74.3, K90.0, D59.1, D69.3, G61.0, G35.0, G35.1, G35.10, G35.11, G35.2, G35.20, G35.21, G35.3, G35.30, G35.31, G35.9, G36.0, G36.1, G36.8, G36.9, G37.0, G37.1, G37.2, G37.5, G37.8, G37.9, G70.0, M45.0, M45.00, M45.01, M45.02, M45.03, M45.04, M45.05, M45.06, M45.07, M45.08, M45.09, M33.1, M08.1, M08.10, M08.11, M08.12, M08.13, M08.14, M08.15, M08.16, M08.17, M08.18, M08.19, M32.1, M32.8, M32.9, M35.3, M33.2, M05.0, M05.00, M05.01, M05.02, M05.03, M05.04, M05.05, M05.06, M05.07, M05.08, M05.09, M05.8, M05.80, M05.81, M05.82, M05.83, M05.84, M05.85, M05.86, M05.87, M05.88, M05.89, M05.9, M05.90, M05.91, M05.92, M05.93, M05.94, M05.95, M05.96, M05.97, M05.98, M05.99, M06.0, M06.00, M06.01, M06.02, M06.03, M06.04, M06.05, M06.06, M06.07, M06.08, M06.09, M06.2, M06.20, M06.21, M06.22, M06.23, M06.24, M06.25, M06.26, M06.27, M06.28, M06.29, M06.3, M06.30, M06.31, M06.32, M06.33, M06.34, M06.35, M06.36, M06.37, M06.38, M06.39, M06.4, M06.40, M06.41, M06.42, M06.43, M06.44, M06.45, M06.46, M06.47, M06.48, M06.49, M06.8, M06.80, M06.81, M06.82, M06.83, M06.84, M06.85, M06.86, M06.87, M06.88, M06.89, M06.9, M06.90, M06.91, M06.92, M06.93, M06.94, M06.95, M06.96, M06.97, M06.98, M06.99, M12.3, M12.30, M12.31, M12.32, M12.33, M12.34, M12.35, M12.36, M12.37, M12.38, M12.39, D86.0, D86.1, D86.2, D86.3, D86.8, D86.9, M34.0, M34.1, M34.8, M34.9, M31.6, M35.2, M30.1, M31.0, M30.3, D89.1, M31.9, M30.0, M30.2, M30.8, M31.4, M31.3 |

# RECORD statement

**Supplementary table S13** The RECORD statement – checklist of items, extended from the STROBE statement, which should be reported in observational studies using routinely collected health data

|  | **Item No.** | **STROBE items** | **Location in manuscript where items are reported** | **RECORD items** | **Location in manuscript where items are reported** |
| --- | --- | --- | --- | --- | --- |
| **Title and abstract** | | | | | |
|  | 1 | (a) Indicate the study’s design with a commonly used term in the title or the abstract (b) Provide in the abstract an informative and balanced summary of what was done and what was found | (a) Abstract, Methods  (b) Abstract, Methods and Abstract, Findings | RECORD 1.1: The type of data used should be specified in the title or abstract. When possible, the name of the databases used should be included.  RECORD 1.2: If applicable, the geographic region and timeframe within which the study took place should be reported in the title or abstract.  RECORD 1.3: If linkage between databases was conducted for the study, this should be clearly stated in the title or abstract. | Title and Abstract  Title and Abstract  Not applicable |
| **Introduction** | | | | | |
| Background rationale | 2 | Explain the scientific background and rationale for the investigation being reported | Introduction, paragraph 1-3 |  | Introduction, paragraph 1-3 |
| Objectives | 3 | State specific objectives, including any prespecified hypotheses | Introduction, last paragraph |  | Introduction, last paragraph |
| **Methods** | | | | | |
| Study Design | 4 | Present key elements of study design early in the paper | Methods, subsection “Study design and data” |  |  |
| Setting | 5 | Describe the setting, locations, and relevant dates, including periods of recruitment, exposure, follow-up, and data collection | Methods, subsections “Study design and data” and “COVID-19 and control cohorts” |  |  |
| Participants | 6 | *(a) Cohort study* - Give the eligibility criteria, and the sources and methods of selection of participants. Describe methods of follow-up  *Case-control study* - Give the eligibility criteria, and the sources and methods of case ascertainment and control selection. Give the rationale for the choice of cases and controls  *Cross-sectional study* - Give the eligibility criteria, and the sources and methods of selection of participants  *(b) Cohort study* - For matched studies, give matching criteria and number of exposed and unexposed  *Case-control study* - For matched studies, give matching criteria and the number of controls per case | (a) Methods, subsections “COVID-19 and control cohorts”  (b) Methods, subsections “COVID-19 and control cohorts”, Figure 1 | RECORD 6.1: The methods of study population selection (such as codes or algorithms used to identify subjects) should be listed in detail. If this is not possible, an explanation should be provided.  RECORD 6.2: Any validation studies of the codes or algorithms used to select the population should be referenced. If validation was conducted for this study and not published elsewhere, detailed methods and results should be provided.  RECORD 6.3: If the study involved linkage of databases, consider use of a flow diagram or other graphical display to demonstrate the data linkage process, including the number of individuals with linked data at each stage. | Methods, subsections “COVID-19 and control cohorts”, Figure 1  Not applicable  Not applicable |
| Variables | 7 | Clearly define all outcomes, exposures, predictors, potential confounders, and effect modifiers. Give diagnostic criteria, if applicable. | Methods, subsections “Health outcomes” and “COVID-19 and control cohorts”,  Supplementary material sections D-F | RECORD 7.1: A complete list of codes and algorithms used to classify exposures, outcomes, confounders, and effect modifiers should be provided. If these cannot be reported, an explanation should be provided. | Methods, subsections “Health outcomes” and “COVID-19 and control cohorts”,  Supplementary material sections D-F |
| Data sources/ measurement | 8 | For each variable of interest, give sources of data and details of methods of assessment (measurement).  Describe comparability of assessment methods if there is more than one group | Methods, subsection “Study design and data” |  |  |
| Bias | 9 | Describe any efforts to address potential sources of bias | Methods, subsection “COVID-19 and control cohorts” |  |  |
| Study size | 10 | Explain how the study size was arrived at | Methods, subsection “COVID-19 and control cohorts”, Figure 1 |  |  |
| Quantitative variables | 11 | Explain how quantitative variables were handled in the analyses. If applicable, describe which groupings were chosen, and why | Methods, subsections “Health outcomes” and “Statistical analyses” |  |  |
| Statistical methods | 12 | (a) Describe all statistical methods, including those used to control for confounding  (b) Describe any methods used to examine subgroups and interactions  (c) Explain how missing data were addressed  (d) *Cohort study* - If applicable, explain how loss to follow-up was addressed  *Case-control study* - If applicable, explain how matching of cases and controls was addressed  *Cross-sectional study* - If applicable, describe analytical methods taking account of sampling strategy  (e) Describe any sensitivity analyses | (a) Methods, subsection “Statistical analyses”  (b) Methods, subsection “Statistical analyses”  (c) Not applicable  (d) Methods, subsection “Statistical analyses”,  Results, subsection “Descriptives of the COVID-19 and control cohorts”, Table 1  Methods, subsection “Statistical analyses” |  |  |
| Data access and cleaning methods |  | .. |  | RECORD 12.1: Authors should describe the extent to which the investigators had access to the database population used to create the study population.  RECORD 12.2: Authors should provide information on the data cleaning methods used in the study. | Methods, subsection “Study design and data” |
| Linkage |  | .. |  | RECORD 12.3: State whether the study included person-level, institutional-level, or other data linkage across two or more databases. The methods of linkage and methods of linkage quality evaluation should be provided. | Not applicable |
| **Results** | | | | | |
| Participants | 13 | (a) Report the numbers of individuals at each stage of the study (*e.g.*, numbers potentially eligible, examined for eligibility, confirmed eligible, included in the study, completing follow-up, and analysed)  (b) Give reasons for non-participation at each stage.  (c) Consider use of a flow diagram | Methods, subsection “COVID-19 and control cohorts”, Figure 1  Figure 1  Figure 1 | RECORD 13.1: Describe in detail the selection of the persons included in the study (*i.e.,* study population selection) including filtering based on data quality, data availability and linkage. The selection of included persons can be described in the text and/or by means of the study flow diagram. | Methods, subsection “COVID-19 and control cohorts”, Figure 1 |
| Descriptive data | 14 | (a) Give characteristics of study participants (*e.g.*, demographic, clinical, social) and information on exposures and potential confounders  (b) Indicate the number of participants with missing data for each variable of interest  (c) *Cohort study* - summarise follow-up time (*e.g.*, average and total amount) | Results, subsection “Descriptives of the COVID-19 and control cohorts”, Table 1 |  | Results, subsection “Descriptives of the COVID-19 and control cohorts”, Table 1 |
| Outcome data | 15 | *Cohort study* - Report numbers of outcome events or summary measures over time  *Case-control study* - Report numbers in each exposure category, or summary measures of exposure  *Cross-sectional study* - Report numbers of outcome events or summary measures | Results, subsections “Overview of relevant health outcomes at three and twelve months follow-up”, and “Incidence of health outcomes by age group”, Table 2,  Supplementary table S7 |  |  |
| Main results | 16 | (a) Give unadjusted estimates and, if applicable, confounder-adjusted estimates and their precision (e.g., 95% confidence interval). Make clear which confounders were adjusted for and why they were included  (b) Report category boundaries when continuous variables were categorized  (c) If relevant, consider translating estimates of relative risk into absolute risk for a meaningful time period | Results, subsections “Overview of relevant health outcomes at three and twelve months follow-up”, and “Incidence of health outcomes by age group”, Table 2,  Supplementary table S7 |  |  |
| Other analyses | 17 | Report other analyses done—e.g., analyses of subgroups and interactions, and sensitivity analyses | Supplementary tables S1-S6 |  |  |
| **Discussion** | | | | | |
| Key results | 18 | Summarise key results with reference to study objectives | Discussion subsection “Interpretation of results” paragraph 1-5 |  |  |
| Limitations | 19 | Discuss limitations of the study, taking into account sources of potential bias or imprecision. Discuss both direction and magnitude of any potential bias | Discussion, subsection “Strengths and limitations” | RECORD 19.1: Discuss the implications of using data that were not created or collected to answer the specific research question(s). Include discussion of misclassification bias, unmeasured confounding, missing data, and changing eligibility over time, as they pertain to the study being reported. | Discussion, subsection “Strengths and limitations” |
| Interpretation | 20 | Give a cautious overall interpretation of results considering objectives, limitations, multiplicity of analyses, results from similar studies, and other relevant evidence | Discussion subsection “Interpretation of results” paragraphs 6-8 |  |  |
| Generalisability | 21 | Discuss the generalisability (external validity) of the study results | Discussion, subsection “Strengths and limitations” |  |  |
| **Other Information** | | | | | |
| Funding | 22 | Give the source of funding and the role of the funders for the present study and, if applicable, for the original study on which the present article is based | Acknowledgements |  |  |
| Accessibility of protocol, raw data, and programming code |  |  | Data availability statement | RECORD 22.1: Authors should provide information on how to access any supplemental information such as the study protocol, raw data, or programming code. | Data availability statement |

*Reference: Benchimol EI, Smeeth L, Guttmann A, Harron K, Moher D, Petersen I, Sørensen HT, von Elm E, Langan SM, the RECORD Working Committee. The REporting of studies Conducted using Observational Routinely-collected health Data (RECORD) Statement. *PLoS Medicine* 2015; in press.

*Checklist is protected under Creative Commons Attribution ([CC BY](http://creativecommons.org/licenses/by/4.0/)) license.
